# Interpreting Microbiome Signatures with MicrobiomeNet

**DOI:** 10.1002/cpz1.70338

**Published:** 2026-02-28

**Authors:** Yao Lu, Khoi Nguyen Nguyen, Jianguo Xia

**Affiliations:** ^1^ Department of Microbiology and Immunology McGill University Montreal Canada; ^2^ Institute of Parasitology McGill University Montreal Canada

**Keywords:** community functions, genome‐scale metabolic model, metabolic interaction, microbiome

## Abstract

MicrobiomeNet (https://microbiomenet.com) is a web‐based platform developed to provide functional insights into microbiome signatures using genome‐scale metabolic models (GEMs). It currently hosts 12,400 GEMs and around 6 million microbial signatures. Users can start by searching microbes, metabolites, genes, or enzymes, and perform common tasks such as to characterize the metabolic capacity for a given microbe, to explore known microbial associations, as well as to understand potential metabolic interactions. This book chapter provides practical, step‐by‐step instructions for navigating MicrobiomeNet to obtain functional insights into individual microbes or microbial association networks. © 2026 The Author(s). *Current Protocols* published by Wiley Periodicals LLC.

**Basic Protocol 1**: Characterizing the Metabolic Profile of a Microbe of Interest

**Basic Protocol 2**: Elucidating Metabolic Interactions from Microbial Associations

**Basic Protocol 3**: Analyzing Carbohydrate‐Utilization Pathways to Explain Co‐Responsive Taxa

**Basic Protocol 4**: Identifying Novel Deoxycholic Acid‐Producing Gut Microbes

**Basic Protocol 5**: Assessing the *Faecalibacterium prausnitzii*–*Coprococcus* Relationship

## Introduction

The growing number of microbiome studies has revealed numerous microbial signatures associated with various pathophysiological and environmental conditions. Understanding their functions and community interactions is integral for translational applications. To address this need, we developed MicrobiomeNet by leveraging genome‐scale metabolic models (GEMs) (Lu et al., 2025)

A GEM serves as a comprehensive digital blueprint of a microbe's metabolic potential. These models can be constructed using two primary strategies: the traditional bottom‐up approach, which involves iteratively adding reactions to enable growth on different media, or the top‐down approach, which infers metabolic capabilities directly from genetic evidence (Machado et al., 2018). Beyond describing the metabolic capacity of individual organisms, GEMs can be used to predict their nutritional needs and to infer potential interspecies interactions, such as competition or metabolic complementarity via the reverse ecology framework (Levy & Borenstein, 2013). While GEMs are inherently defined at the strain level, the metabolic potential of a microbial community or higher taxonomic level can be approximated by aggregating the GEMs of its constituent members (Douglas et al., 2020; Lu et al., 2023). Such composite GEMs enable broad overviews of community functions and insights into underlying ecological niches. High‐quality GEMs are now increasingly available, such as AGORA and CarveMe (Heinken et al., 2023; Heinken et al., 2025; Magnúsdóttir et al., 2017; Seaver et al., 2021), providing opportunities to understand microbiome associations and co‐occurrence patterns (Faust et al., 2012; Geistlinger et al., 2024).

Building on these concepts, we developed MicrobiomeNet, a comprehensive platform for investigating microbial metabolic capacities and predicting potential interactions. Users can query a vast collection of >12,400 GEMs and ∼5.8 million microbial signatures. These results can be visually explored as microbial association networks, metabolic pathways, or networks. This book chapter introduces MicrobiomeNet through five Basic Protocols. Basic Protocols 1 and 2 describe general analysis workflows and visualization functions of MicrobiomeNet. Basic Protocols 3–5 demonstrate how to apply MicrobiomeNet in specific biological contexts, including investigating carbohydrate‐utilization pathways under dietary interventions, identifying deoxycholic acid–producing gut microbes, and exploring microbial interactions related to the gut–brain axis.

## Basic Protocol 1 CHARACTERIZING THE METABOLIC PROFILE OF A MICROBE OF INTEREST

The objective of this protocol is to demonstrate how to search and explore the detailed metabolic capacity of a taxon using MicrobiomeNet. Here, we use *Faecalibacterium prausnitzii*, a dominant bacterial species in the human gut. *F. prausnitzii* is widely recognized for its beneficial role in intestinal health, and its abundance is often reduced in inflammatory and metabolic disorders (Miquel et al., 2013). *F. prausnitzii* produces bioactive molecules such as butyrate and engages in active cross‐feeding interactions with other gut microbes. *Bacteroides* species such as *B. adolescentis* and *B. fragilis* can release short‐chain carbon substrates that are utilized by *F. prausnitzii*, resulting in significantly higher butyrate production (Lindstad et al., 2021; Rios‐Covian et al., 2015). In this protocol, we show how to examine the detailed pathways and reactions underlying this function, how butyrate production is achieved and integrated into the broader metabolic network of *F. prausnitzii*, and how potential cross‐feeding within the gut microbial community occurs.

### Necessary Resources

#### Hardware

A computer with internet access

##### Software

An up‐to‐date web browser such as Google Chrome, Mozilla Firefox, or Safari, with JavaScript enabled (see Internet Resources)

##### Files

None

**Figure 1:**
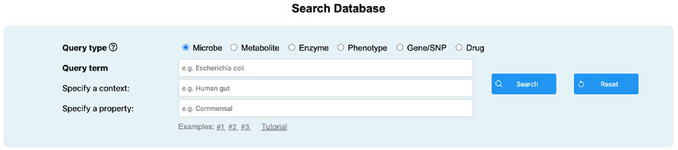
The search bar on the MicrobiomeNet home page is the starting point for initiating a query.

1 Go to the MicrobiomeNet home page (https://microbiomenet.com). Locate the search bar in the middle section of the home page. MicrobiomeNet accepts various search inputs, including microbe, metabolite, enzyme, etc. In this example, leave “Microbe” as the Query Type, type *Faecalibacterium prausnitzii* as the query term, and “Human gut” as the study context, and then click the “Search” button (Figure 1). Alternatively, users can directly choose the first example by clicking “#1” and “Search” to start.
  Understanding microbial functions and interactions requires a context‐dependent approach. MicrobiomeNet facilitates this by offering ∼20 predefined contexts, including Human gut, Human oral, Human respiratory, Soil, Marine, etc. **Table** **1** summarizes GEMs and their associated contexts. Unless otherwise specified, downstream analysis will be conducted within the context specified.

**Table 1:** Distribution of the Context in GEM Collection

Context	Phylum	Genus	Species	Strain
Aquatic Animal	10	39	62	62
Freshwater	18	116	161	162
Human gut	35	469	1465	2111
Human oral	17	83	132	211
Human other	41	406	1161	5679
Human respiratory	4	12	28	28
Human skin	2	4	8	8
Human vagina/urogenital	14	45	62	97
Marine	18	178	303	307
Mouse	10	12	16	16
Mouse gut	11	33	34	37
Mouse other	10	50	58	66
Other/Unknown	48	1232	2581	2629
Plant‐associated	8	54	95	99
Sediment	10	77	100	100
Soil	13	196	420	432
Terrestrial Animal	14	70	129	133
Wastewater	5	22	23	23

**Figure 2:**
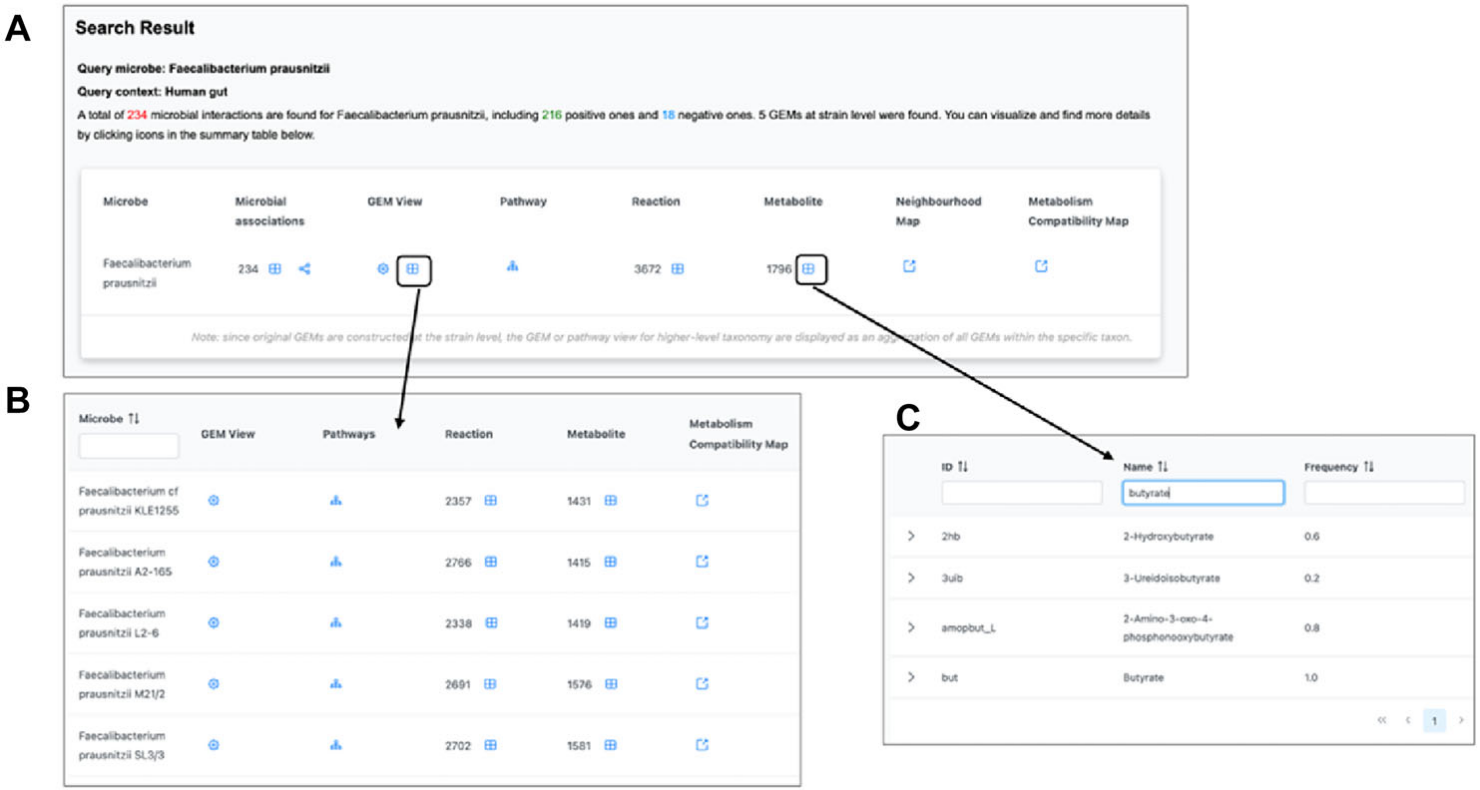
(A) The summary table of search results for the query taxon *F. prausnitzii* and the query context “Human gut”. (B) Five GEMs identified for *F. prausnitzii* in the human gut. (C) Metabolites produced by *F. prausnitzii* in the human gut, with a focus on butyrate.

2 The search result page shows a table with links to metabolites, reactions, pathways, and GEM view, as well as known associations for *F. prausnitzii* (Figure 2**A**).

**Figure 3:**
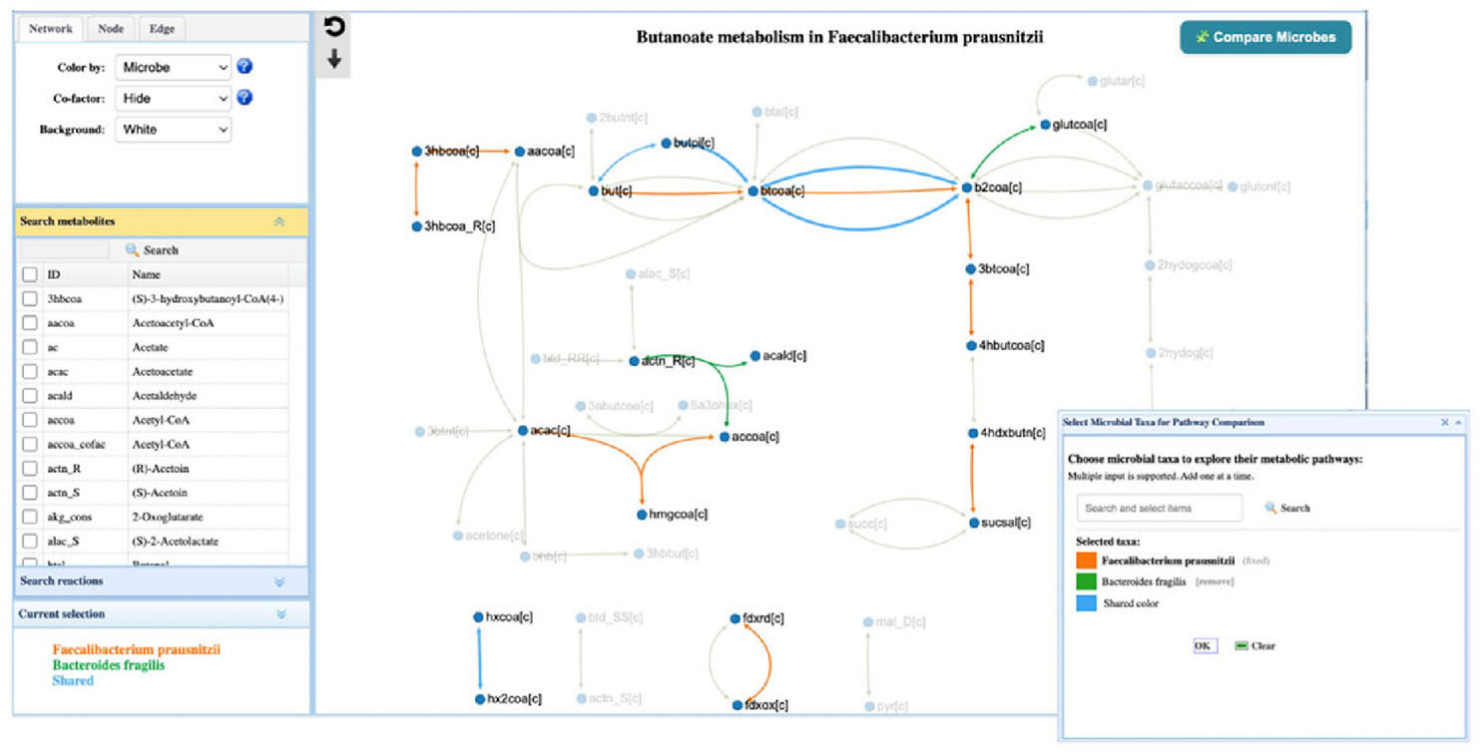
Potential metabolic cross feeding between *F*. *prausnitzii* and *B. fragilis* in Butanoate metabolism. The color legend for each species is shown in the lower‐left panel. Further comparisons or updates can be performed by clicking the “Compare Microbes” button on the top right to customize the species and their corresponding colors. The left control panel allows users to adjust the pathway visualization and highlight specific metabolites or reactions of interest.

3 By default, MicrobiomeNet aggregates strain‐level GEMs into a composite GEM model to represent the query species *F. prausnitzii*. Click the table icon under the column “GEM view” to find more information about the underlying GEMs (Figure 2**B**).
  The composite GEM is built from the full set of models attributed to the given taxon in the selected context. In this case, five GEMs were retrieved under the species F. prausnitzii within the context of the Human gut microbiome. This composite is used by default for downstream analysis, though users can choose to proceed with only a specific, individual GEM
4 Click the table icon under the “Metabolite” column to view all metabolites involved in the metabolism of *F. prausnitzii*. Under the “Name” column, enter “butyrate” to search the table. The frequency value of 1 indicates that all five GEMs belonging to *F. prausnitzii* in the human gut can produce butyrate (Figure 2**C**).
5 Click “Search Result” on the top navigation menu to go back to the summary page. Click the pathway icon to view all the pathways in *F. prausnitzii*. Scroll down to locate the “Butanoate metabolism” pathway (or use the Search box on the top right corner). Click the corresponding pathway thumbnail to navigate to the pathway visualization page.
6 The central panel displays the butanoate metabolism pathway, encompassing all known reactions within the current context (e.g., the human gut). Reactions present in *F. prausnitzii* are highlighted (Figure 3). Users can interactively explore this pathway to understand the metabolic potential.
  The top‐left control panel is used to adjust the network's appearance, including the color and size of nodes and edges, as well as the background. By default, the pathway displays only the main substrates and products for clarity. Users can toggle the display of cofactors to gain deeper insights into specific reactions. By setting the node mode to “Drag”, users can manually adjust metabolites within the network to improve visualization.
  Metabolites and reactions involved in the metabolism are listed in the middle‐left accordion panel. Click an item in this panel to highlight the corresponding metabolite or reaction in the pathway. For example, typing “butyrate” in the search box or scrolling to check it will highlight the metabolite in the network. This allows users to easily identify its associated reactions, such as the butyryl‐CoA:acetate CoA‐transferase step, which is central to the butyrate pathway in F. prausnitzii.

**Figure 4:**
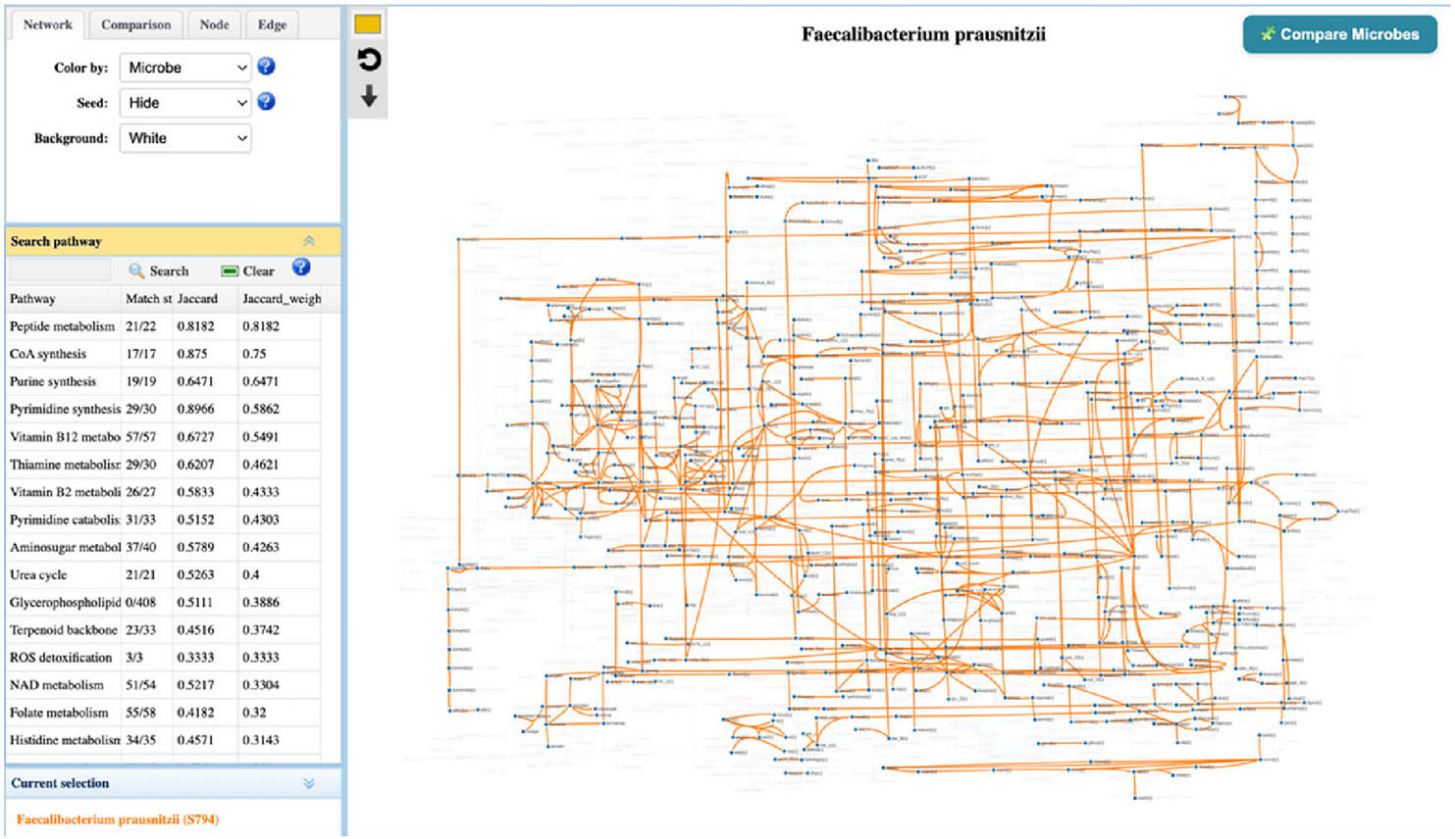
Detailed view of metabolic map for *F. prausnitzii* providing an overview of its reconstructed metabolism. The network represents the combined reactions from the five identified GEMs. The pathway panel on the middle‐left displays both standard and weighted Jaccard indices, illustrating the coverage and distribution of *F. prausnitzii*’s metabolic functions across each pathway.

7 To showcase how to compare pathways between two microbes, we would like to compare this pathway between *Bacteroides fragilis* and *F. prausnitzii*. Click the “Compare Microbes” button located at the top‐right corner to open the dialog. Within this panel, two microbial taxa can be added using the search box. The selected species are displayed in distinct colors for visualization, and users can modify them by clicking the color box. For example, type *Bacteroides fragilis* in the search box and assign it a color different from *F. prausnitzii*. The color of the reference taxon can also be adjusted. After selection, click the “OK” button to view the highlighted reactions of the selected microbes in the pathway (Figure 3).
  B. fragilis is a well‐known primary degrader of complex carbohydrates, generating simple fermentation products that serve as substrates for secondary fermenters such as F. prausnitzii (Louis & Flint, 2009). In this example, B. fragilis provides precursor metabolites that F. prausnitzii can utilize for butyrate production through butyryl‐CoA pathways. F. prausnitzii can efficiently convert these substrates into butyrate via the butyryl‐CoA:acetate CoA‐transferase route, which is its dominant butyrate‐producing mechanism. Their cooperative cross‐feeding in the healthy gut microbiome is well documented (Culp & Goodman, 2023).
8 Return to the “Search Result” page and click the “GEM view” icon to explore the overall metabolism of *F. prausnitzii*. The GEM map (Figure 4) highlights the metabolic capacity through a combined visualization of reactions, metabolites, and pathways. This map corresponds to the composite GEM generated from the five GEM models identified in the selected context (i.e., the human gut). The top‐left control panel is similar to the one on the pathway page and can be used to adjust the network's appearance. Details of the “Seed option” and the comparison functions will be introduced in Step 10 of Protocol 2. All pathways associated with *F. prausnitzii* are listed in the middle‐left pathway panel, where users can search for and highlight pathways of interest on the central panel. For visual clarity, very large pathways are not displayed directly on this page. In this case, users can follow the provided hyperlink to open the corresponding pathway page for further analysis.

9 The pathway table offers standard and weighted Jaccard indices for each pathway to quantify the overlap between *F. prausnitzii* and the total reaction set of the selected context (e.g., human gut). The standard Jaccard index reflects reaction coverage based solely on binary presence or absence, whereas the weighted Jaccard index incorporates reaction abundance. *F. prausnitzii* shows high standard Jaccard values in essential biosynthetic functions, such as pyrimidine/purine synthesis, peptide metabolism, and vitamin production (e.g., B2, B12, thiamine). This confirms that the species possesses a complete core metabolic framework required for growth and maintenance. However, the weighted Jaccard indices reveal nuances in how these pathways are structured compared to the overall gut community. High weighted scores in peptide metabolism indicate that *F. prausnitzii* uses standard, widely shared reactions. In contrast, pathways like pyrimidine and CoA synthesis show a drop in the weighted index despite high coverage. This suggests that while *F. prausnitzii* performs these functions, it utilizes a distinct set of reactions or enzymes compared to the average gut microbe, highlighting a species‐specific metabolic strategy.
  Low Jaccard indices in pathways such as butanoate metabolism, the glyoxylate cycle, sulfur metabolism, and several amino‐acid degradation routes indicate that F. prausnitzii shares relatively few reactions with the broader gut microbiome in these functions. Furthermore, the extremely low weighted Jaccard indices (often below 0.15 and occasionally <0.05) suggest not only limited reaction overlap but also strongly divergent reaction‐frequency patterns. This implies that even when specific reactions are conserved, their abundance or utilization in F. prausnitzii differs significantly from the community average. This combination of low coverage and distinct distribution reflects high metabolic specialization, consistent with pathways that are uniquely regulated or indicative of a complementary rather than competitive ecological role.

In Protocol 1, we used GEMs to validate the butyrate‐producing capacity of *F. prausnitzii* and identify potential cross‐feeding with *B. fragilis*. These results underscore MicrobiomeNet's power to characterize specific microbes and metabolites via advanced model integration and visual analytics.

## Basic Protocol 2 ELUCIDATING METABOLIC INTERACTIONS FROM MICROBIAL ASSOCIATIONS

MicrobiomeNet houses a comprehensive collection of literature‐derived microbial associations. However, translating these statistical co‐occurrence patterns into potential functional interactions remains a key challenge. GEMs can help address this by detailing microbial metabolism and inferring nutritional requirements. By comparing their profiles, we can assess potential metabolic interactions, such as competition or cross‐feeding. MicrobiomeNet supports global overviews and detailed visual assessment of potential metabolic interactions. The objective of this protocol is to demonstrate how to extract known associations for a specific taxon and elucidate their mechanistic basis through GEM‐driven metabolic analysis.

### Necessary Resources

#### Hardware

A computer with internet access

##### Software

An up‐to‐date web browser such as Google Chrome, Mozilla Firefox, or Safari, with JavaScript enabled (see Internet Resources)

##### Files

None

**Figure 5:**
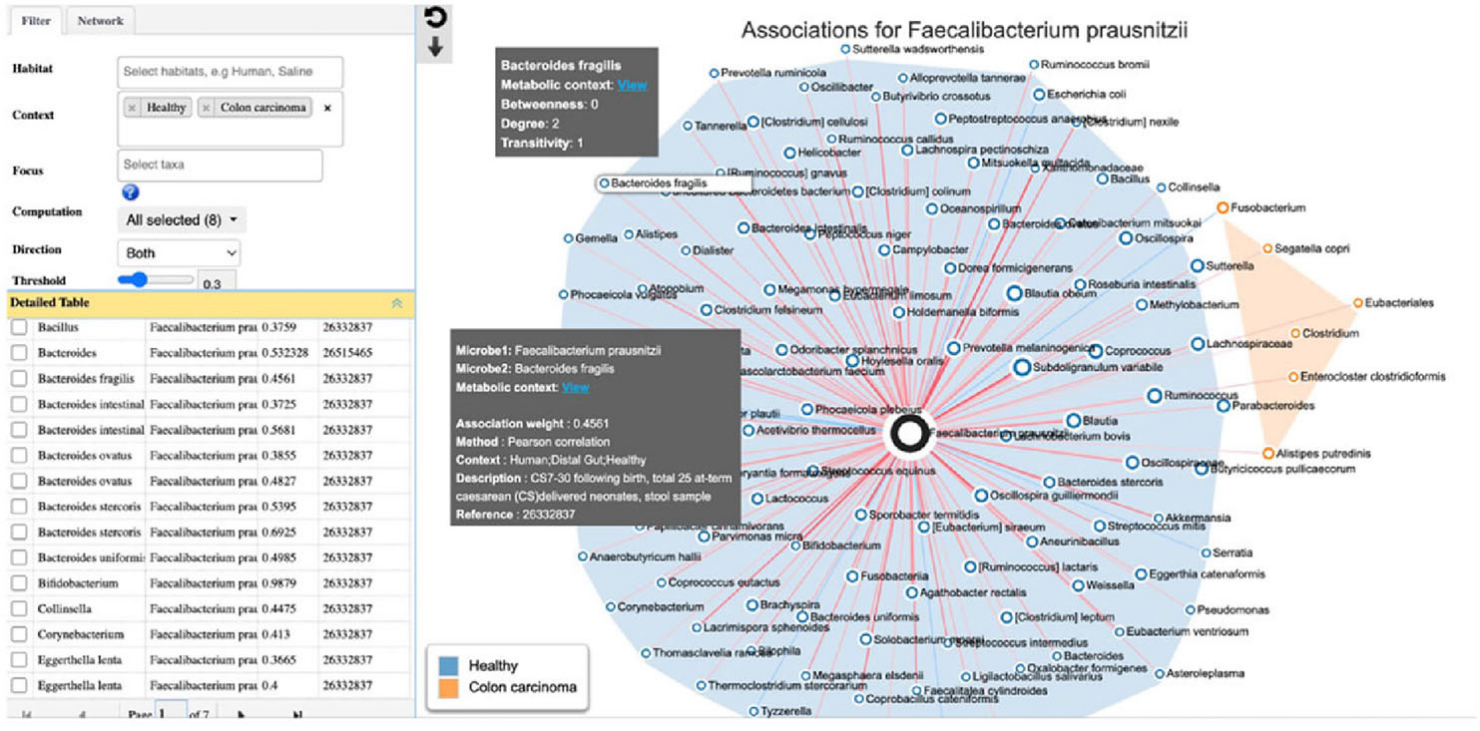
The microbial association network found for *F. prausnitzii*. The top‐left panel allows users to adjust the network settings, including the selection of specific contexts and the overall appearance of the visualization. Users can also view or search the correlation table (middle left) to highlight corresponding edges in the network. Red edges indicate positive correlations, while blue edges represent negative correlations. Clicking any node or edge displays detailed information in a grey tooltip. To explore the metabolism of a specific microbe or the metabolic interaction between a pair of microbes, click the “View” hyperlink within the tooltips to navigate to the metabolic network page.

1 Go to the MicrobiomeNet home page (https://microbiomenet.com). Locate the search bar in the middle section of the home page. Type *Faecalibacterium prausnitzii* as query term, and “Human gut” as query context (or directly choose the first example by clicking “#1”). Click the “Search” button.
2 A total of 234 statistical associations of *F. prausnitzii* found in our database are related to the human gut. Click the table icon under the “Association” column to view the details. The weight, method, and context are listed in the result table. As mentioned above, *Bacteroides* species are reported to interact with *F. prausnitzii* in the human gut. Under the “Taxon2” column, enter “Bacteroides” in the search box to retrieve related associations identified in the current context. All reported associations are positive, with moderate statistical significance. Therefore, further exploring their metabolic potential is essential to reveal the true biological relationships.
  All included statistics were reported as significant in their original studies. However, due to the heterogeneity in analytical methods and normalization procedures, direct numerical comparison between studies is generally not feasible. Consequently, identical microbial pairs observed across different studies or conditions are listed as separate entries. These statistics are provided to facilitate the assessment of broad trends and reproducibility across studies, rather than for direct quantitative comparison.
3 Go back to the “Search Result” page and click the network icon for “Microbial associations”. This will open the page for the microbial association network associated with human gut *F. prausnitzii* (Figure 5). The query microbe is highlighted as a big black ring in the center. Node colors and colored backgrounds indicate different study groups.

**Figure 6:**
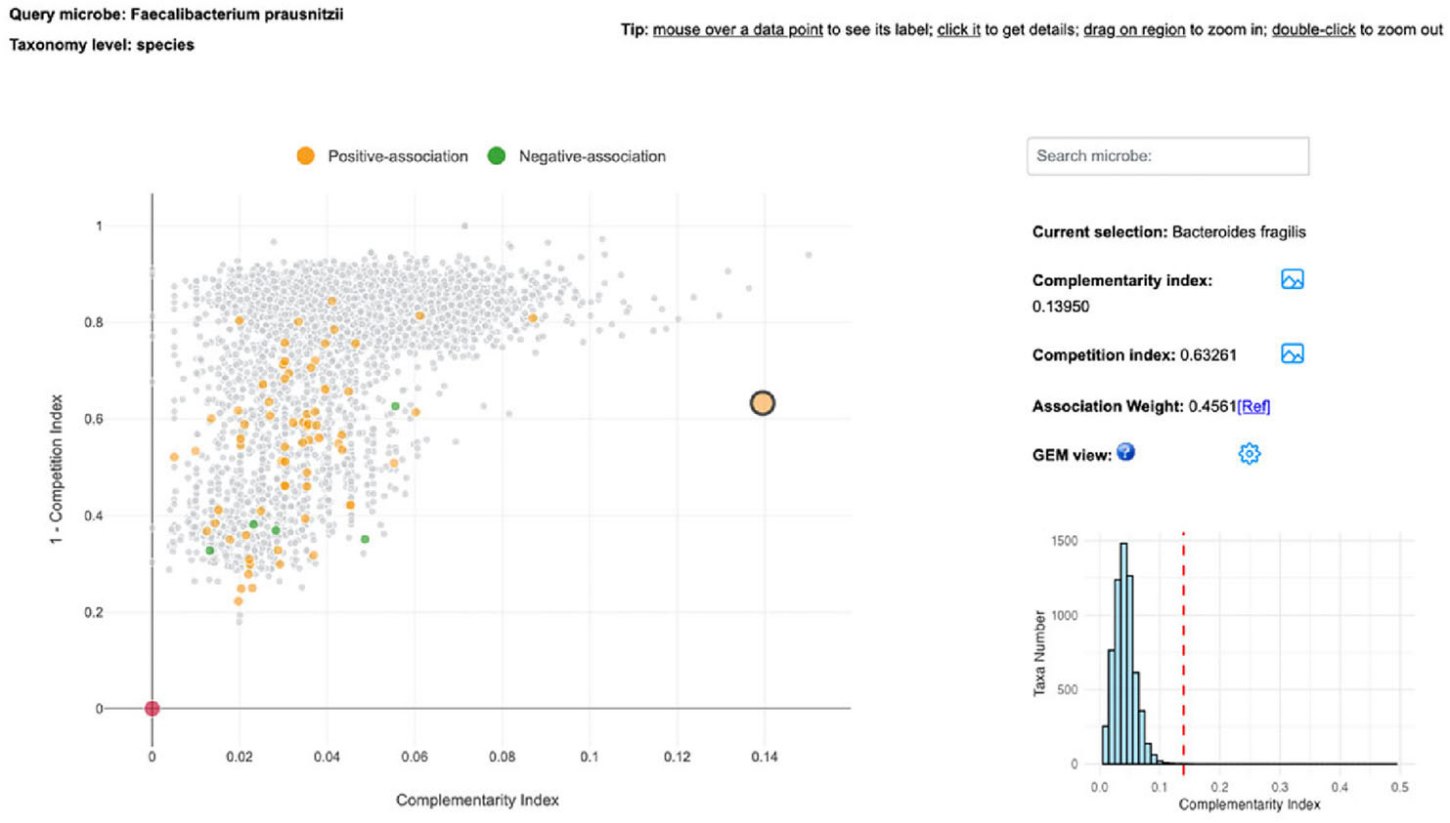
The Metabolism Compatibility Map showing the overall distribution of complementarity and competition indices for all microbes relative to *F. prausnitzii. B. fragilis* shows one of the highest complementarity indices with *F. prausnitzii*, indicating strong metabolic complementarity. Clicking on any point displays detailed information for the selected microbe on the right. Users can further view the distribution of each index by clicking on the corresponding icon, as shown in the histogram for the complementarity index. Clicking the GEM‐view icon will open the metabolic network map, enabling direct comparison of metabolism between the selected microbe and *F. prausnitzii*.

4 On the top left, users can further refine the network by updating the study group, the taxonomic level, as well as the method, threshold, and direction of the associations. Currently, few published studies provide associations at the strain level. In this example, we limited the context to “Healthy” and “Colon carcinoma” to serve as an example to provide a contrast for interpreting association patterns of *F. prausnitzii*. Click the “Update” button to confirm the change. As shown in Figure 5, most of the associations of *F. prausnitzii* are present in the healthy gut, and there is no overlap in correlations between these two study backgrounds.
5 Users can highlight and search any association of interest in the bottom‐left panel. For example, searching for *B. fragilis* highlights its connection with *F. prausnitzii* in the association network. The blue node color indicates that this relationship has been observed in the healthy gut microbiome. The red edge indicates a published positive correlation between the two species, while the correlation index of 0.456 represents a moderate association strength. Both literature evidence and pathway analysis suggest that this association is biologically meaningful. For instance, *Subdoligranulum* species are closely related to *F. prausnitzii*, suggesting they may be promising targets for further mechanistic investigation (del Chierico et al., 2015).
6 To learn more about a specific association, click on either the nodes or the edges in the network. For instance, click the *B. fragilis* node to view its network properties and metabolic context. A tooltip will appear showing key topological metrics, including betweenness, degree, and transitivity, which describe the node's connectivity and role within the association network. By clicking the “View” link on the tooltip, users can navigate to the GEM map to explore the metabolism of the selected taxon (covered in Steps 9‐10).
  Click the edge connecting two microbes to view detailed information about their association. The pop‐up displays key information, including the association weight, correlation method, biological context, and reference source.
7 From Steps 3–6, users obtain an overview of all reported associations for *F. prausnitzii* within the selected context. In many cases, the number of statistical correlations can be large, e.g., over 200 in this example, with a wide range of correlation values. To further evaluate which of these associations are more likely to represent true biological interactions from a metabolic perspective, go back to the “Search Result” page and click the hyperlink icon to visit the “Metabolism Compatibility Map” page.
8 The Metabolism Compatibility Map is shown as a scatter plot integrating the competition and the complementarity information (Figure 6). The colored dots represent taxa reported to be associated with *F. prausnitzii*, with orange dots indicating a positive correlation and green dots indicating a negative correlation. An orange dot stands out as it is located on the right side of the scatter plot, suggesting a high complementarity with *F. prausnitzii*. Hovering over the node shows that it represents *B. fragilis*. Click on the node to view its detailed information. By further clicking the picture icon next to the indices, users can view the ranking of *B. fragilis* across all other species in relation to *F. prausnitzii*. *B. fragili*s ranks among the top species in the complementarity index, while it has a relatively median to low competition index, confirming the previous reports and the pathway‐level observations.
  MicrobiomeNet utilizes seed metabolites—essential nutrients that must be acquired exogenously—to compute competition and complementarity indices. The competition index is defined as the similarity between the seed metabolite profiles of two microbes. In contrast, the complementarity index quantifies the potential of one microbe to synthesize seed metabolites required by another (excluding those required by the producer itself). To facilitate visual comparison, MicrobiomeNet displays (1 ‐ Competition Index), ensuring that both metrics align in the same direction.

**Figure 7:**
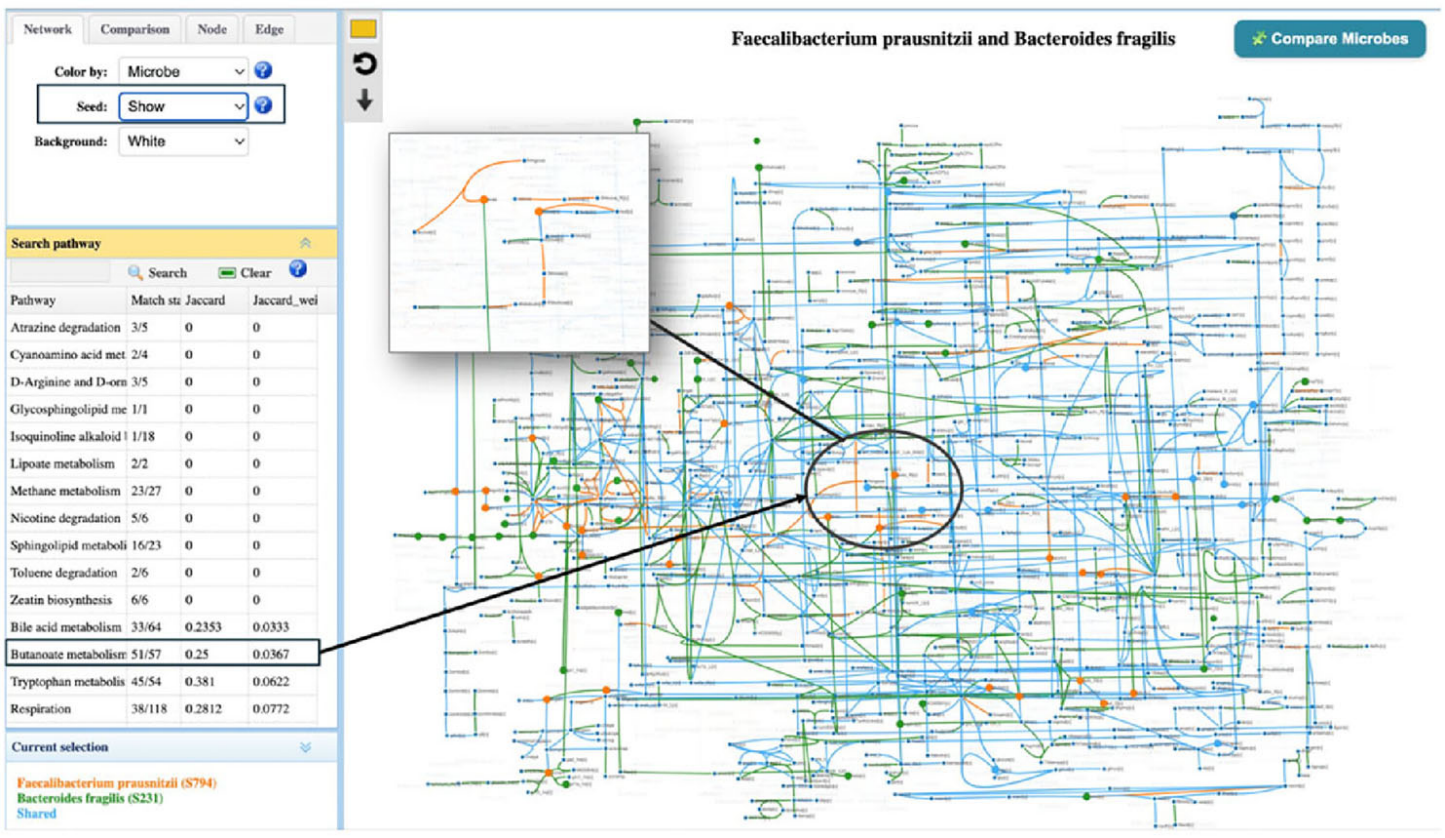
Metabolism comparison for *F. prausnitzii*. and *B. fragilis*. The standard Jaccard index and the weighted Jaccard index for each pathway are listed in the left panel. Lower Jaccard values indicate lower overlap between the two species for the selected pathway. For example, the highlighted butanoate metabolism pathway has an index of 0.25, suggesting lower metabolic competition and higher complementary potential for this pathway. The enlarged pathway shows the interaction within butanoate metabolism.

9 The Metabolism Compatibility Map can be used to explore potential specific associations, such as between *Subdoligranulum variabile* and *F. prausnitzii*, which was found to be highly statistically associated in several studies. However, no direct evidence of their mechanical interaction has been reported in previous studies. Searching for *Subdoligranulum variabile* in the Metabolism Compatibility Map, we find moderate complementarity and a relatively high competition index. From a metabolic interaction perspective, these results suggest that the observed positive correlation is difficult to mechanistically validate.
10 Following Step 6, click “View” on the edge, or in Step 9, click the GEM view icon, to open the GEM map to examine the metabolism in detail. The overall layout of the GEM map is similar to that of the Pathway Visualization page, providing a consistent user experience (Figure 7). The comparative metabolism between the two species is displayed in the central panel. On the left control panel, users can further customize the edge, node, and background. Update the colors to align with the color settings used in Protocol 1. One specific feature is the “Seed” option. By toggling “Seed”, users can visualize the overlap of required metabolites as well as potential cross‐feeding, thus better understanding potential metabolic competition and complementarity between microbes. The seed metabolites are highlighted with a thick border in the species‐specific color. As shown, *F. prausnitzii* and *B. fragilis* shared a small subset of seed metabolites, suggesting they are not strongly competitive for the same nutrients. They can produce seed metabolites for each other through *Butanoate metabolism* and *Starch and sucrose metabolism*.
  The “Comparison” panel can further support the calculation of competition and complementarity indices between the two species. The overall distributions generated for competition and complementarity indices align with the network observations. The competition index between B. fragilis and F. prausnitzii is moderate to low, indicating limited potential for resource competition. In contrast, the complementarity index ranks among the highest compared to other taxa interacting with F. prausnitzii, supporting the presence of potential mutualistic interactions within the community.

11 Users can search and highlight the pathway through the left “Pathway” panel. The standard and weighted Jaccard indices are displayed in the table, allowing users to examine interactions between two selected taxa.
  The Jaccard index quantifies the extent of metabolic overlap between species, and the weighted index extends this by incorporating reaction frequency within the selected taxon. For instance, per‐pathway Jaccard indices calculated between F. prausnitzii and B. fragilis provide a quantitative measure of their shared metabolic repertoires. A high index (e.g., >0.8 in Peptide metabolism and CoA synthesis) indicates substantial similarity, suggesting potential competition. However, the two indices provide slightly different interpretations for the two pathways. For Peptide metabolism, the weighted Jaccard index is slightly higher, meaning the two taxa not only share many reactions but also have similar reaction‐frequency patterns within this pathway. While CoA synthesis has a standard Jaccard index of 1 (all reactions shared), the weighted index is lower, indicating that the relative frequencies of these reactions differ substantially between the two taxa even though the reaction sets are identical. In contrast, pathways such as Butanoate metabolism exhibit relatively low Jaccard indices and very low weighted index (<0.1), reflecting limited overlap and implying complementary metabolic roles.
  By selecting a pathway from the left panel, users can generally view the detailed metabolic interactions between the two species for that specific pathway within the GEM page. For instance, by highlighting Butanoate metabolism, users can see that B. fragilis provides seed metabolites for F. prausnitzii. However, to understand more specific interactions at the pathway level, click the Link icon for each pathway to navigate to pathway visualization (Step 5 in Basic protocol 1)

Protocol 2 walks users through the MicrobiomeNet pipeline from association analysis to metabolic interaction. These steps show that the statistical correlation between *F. prausnitzii* and *B. fragilis* can at least partially be explained by their metabolic complementarity through the butanoate metabolism pathway. It is important to note that researchers can directly search any microbe of interest to access the Neighborhood Map and Metabolism Compatibility Map for potential metabolic interactions beyond known statistical associations.

## Basic Protocol 3 ANALYZING CARBOHYDRATE‐UTILIZATION PATHWAYS TO EXPLAIN CO‐RESPONSIVE TAXA

This case study builds on a human arabinoxylan intervention that identified co‑responsive taxa (Nguyen et al., 2020). Using MicrobiomeNet, it assesses whether these taxa can utilize the target fiber or its breakdown products and whether shared versus complementary pathways explain their co‑response. By searching genome‐scale metabolic models (GEMs) to retrieve polysaccharide‐degradation pathways, the workflow addresses two questions: (i) can the taxa use the target fiber or its breakdown products, and (ii) do they rely on the same or different pathways—hinting at competition or partnership? This workflow outputs detailed pathway‐level evidence to help interpret relationships between significant taxa and the targeted fiber.

### Necessary Resources

#### Hardware

A computer with internet access

##### Software

An up‐to‐date web browser such as Google Chrome, Mozilla Firefox, or Safari, with JavaScript enabled (see Internet Resources)

##### Files

None

**Figure 8:**
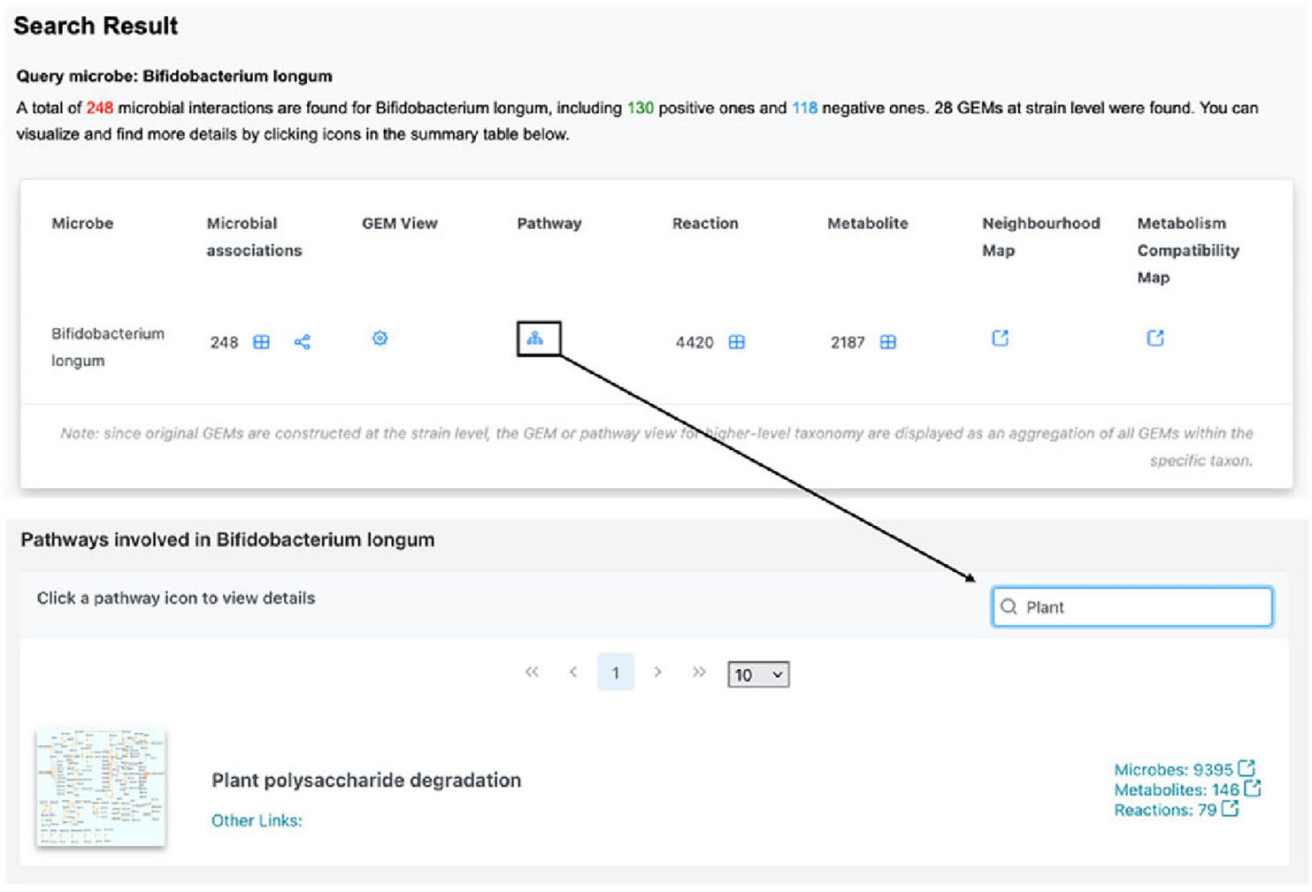
(Top) The summary table of search results for the query taxon *Bifidobacterium longum* without a specific query context. (Bottom) Searching for the plant polysaccharide degradation pathway from the *Bifidobacterium longum* pathway list.

1 Starting up. Go to the MicrobiomeNet home page (https://microbiomenet.com). Search for “*Bifidobacterium longum*” with query context “Human gut” (see Basic Protocol 1 for step‑by‑step details).
2 Pathway search. To evaluate *B. longum*’s capacity on the study fiber, in the result table, click the Pathway icon and use Global search (“Plant”) to locate “Plant polysaccharide degradation” (See step 5 in Basic Protocol 1). Then, click on the thumbnail to navigate to the pathway visualization page (**Figure** **8**).

**Figure 9:**
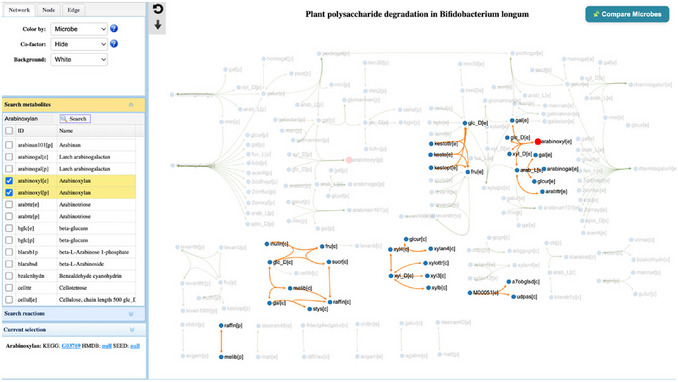
A screenshot of the plant polysaccharide degradation pathway in *Bifidobacterium longum* with a highlighted node of a specific fiber of interest (Arabinoxylan).

3 Exploring the pathway page. This view shows the plant polysaccharide degradation pathway for *B. longum*. Pathways that do not belong to this microbe appear in dim gray, while those belonging to *B. longum* are highlighted in orange (**Figure** **9**).

**Figure 10:**
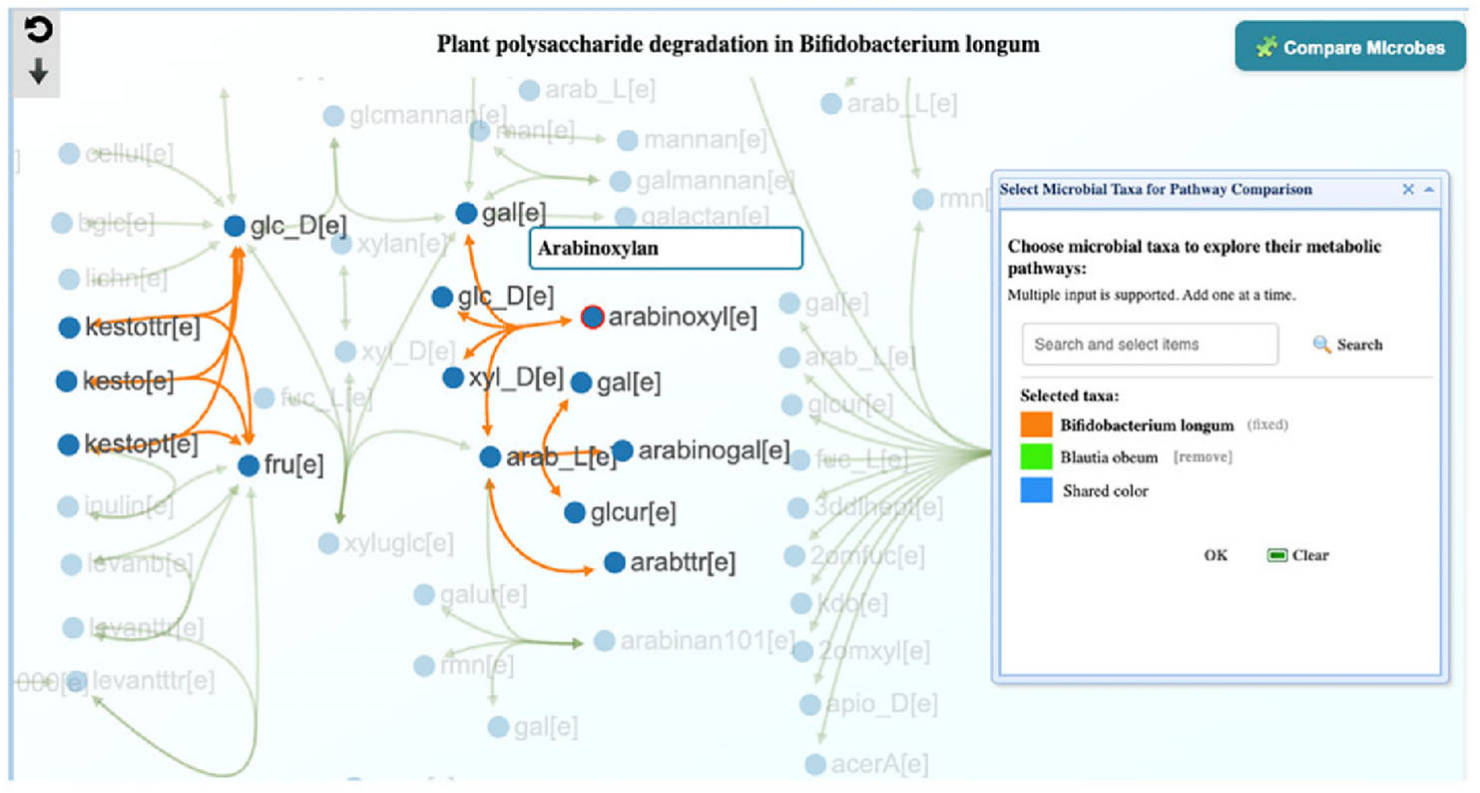
Plant polysaccharide degradation pathway comparison between *Bifidobacterium longum* (orange) and *Blautia obeum* (Green), where shared pathways are blue. The view focuses on arabinoxylan (fiber of interest).

4 Locate the reaction chain for the target fiber. To quickly assess whether *B. longum* can act on the study fiber (arabinoxylan), first check whether reactions involving arabinoxylan exist in its pathway map. On the pathway page, locate the “Search metabolites” panel on the left, type “arabinoxylan” in the Search box, and click Search. The arabinoxylan node (e.g., arabinoxyl[e], where [e] denotes extracellular) will be highlighted in red. Follow the orange edges from arabinoxyl[e] to downstream products (e.g., glc_D[e], xyl_D[e]); the presence of these conversions indicates that *B. longum* can degrade the targeted fiber.
5 Pathway overlay. To identify the different and shared Plant polysaccharide degradation pathways between *B. longum* and *Blautia obeum*, we need to map the pathways of these two bacteria. Click the “Compare Microbes” icon (right corner) to open the “pathway comparison” window by query taxa. In the search box, type “Blautia obeum” and select it from the drop‐down. Click “Search” to confirm the selected taxa (*Bifidobacterium longum* and *Blautia obeum*) and select colors for each bacterium (orange & green) and the shared pathway (blue), then click the “OK” button (**Figure** **10**).

6 Perform pathway comparison. The view shows pathway components shared between *B. longum* and *Blautia obeum*. Orange edges mark segments present only in *Bifidobacterium longum*; blue edges mark shared segments. Assess utilization of the target fiber in both taxa. Check whether both organisms can utilize the target fiber pathway or only one. MicrobiomeNet shows the metabolite ID next to each node. Move the cursor over a node to display an interactive box with the full metabolite name. The letter inside the square bracket indicates the compartment name (**Table** **2**). Please refer to Table 1 for the full name. Here are the IDs and full names of metabolites related to arabinoxylan degradation in B. longum and B. obeum: arabinoxyl = arabinoxylan; glc_D = D‐glucose; xyl_D = aldehydo‐D‐xylose; gal = D‐galactose; arab_L = L‐arabinose; xylan4 = xylan with 4 backbone units and 1 glucose side chain; glcur = D‐glucuronate (**Figure** **10**).

**Table 2:** Compartment IDs and Full Names

ID	Name
c	Cytosol
e	Extracellular space
p	Periplasm

**Figure 11:**
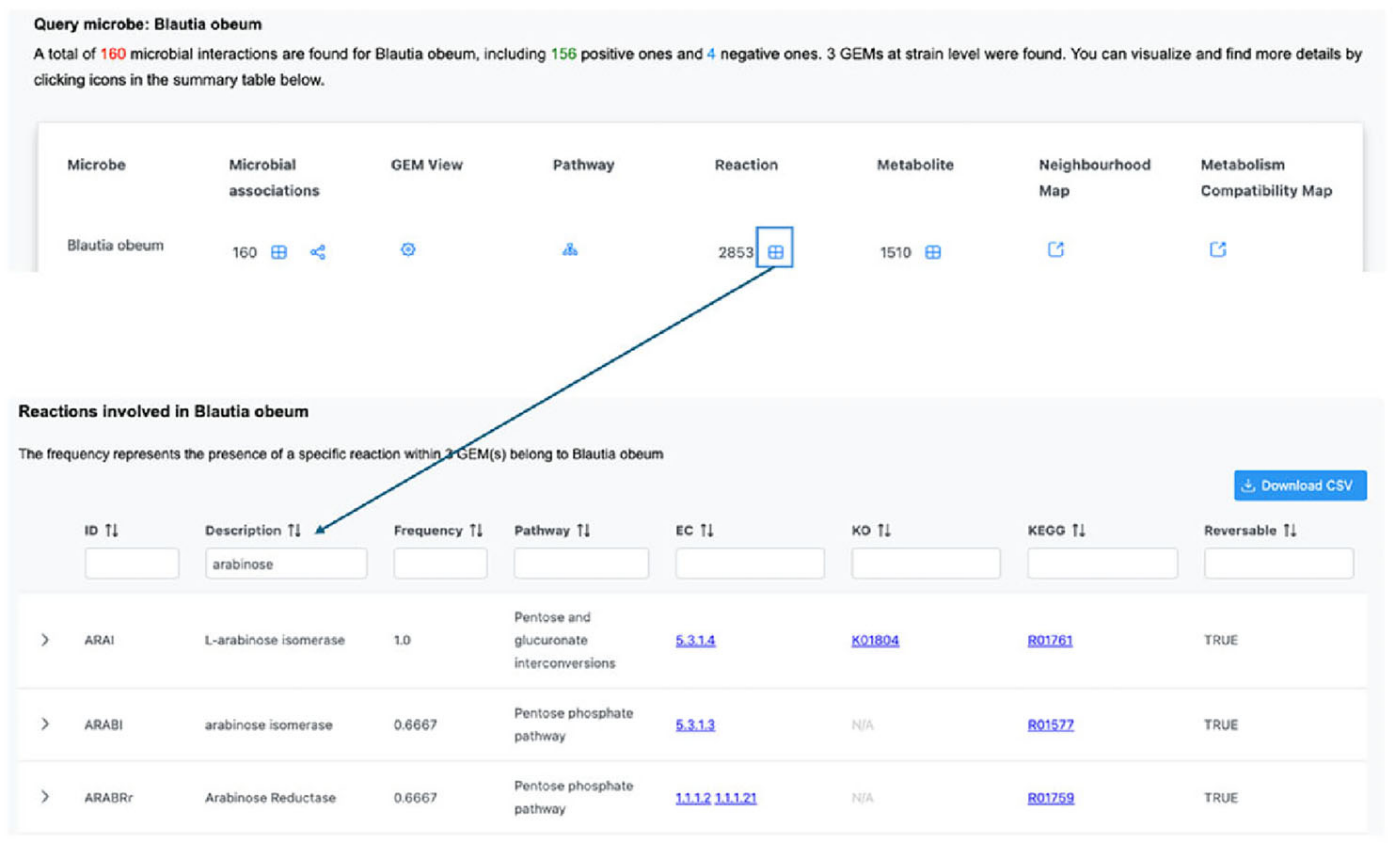
(Top) Summary table of search results for the query taxon *Blautia obeum* without a specific query context. (Bottom) Searching for arabinose‐related reactions and pathways.

7 Identify relevant carbohydrate‐utilization pathways in *Blautia obeum*. In the case study, *B. longum* and *Blautia obeum* clustered in the same response group that showed a strong increase under the arabinoxylan consumption arm. In MicrobiomeNet, *B. longum* is predicted to utilize arabinoxylan, whereas *Blautia obeum* is predicted to utilize xylan (the arabinoxylan backbone) and convert it to D‐glucuronate and D‐xylose. At this point, we can answer the first question: *B. longum* can utilize the target fiber (arabinoxylan), but *Blautia obeum* cannot; thus, their relationship on this substrate is unlikely to be competitive. To further resolve their relationship, examine whether *Blautia obeum* can utilize arabinoxylan‐derived subunits via other pathways (e.g., L‐arabinose, D‐glucose, D‐galactose, D‐xylose, D‐glucuronate). Go to the MicrobiomeNet home page and search for “*Blautia obeum*”. In the summary table, click the Reactions icon to open the sortable reactions table for *Blautia obeum*. In the filter box, type “arabinose” and review the Pathway column to identify any relevant pathways (**Figure** **11**). If present, note the pathway name, then repeat for other arabinoxylan subunits (e.g., xylose, galactose, glucuronate)—skip glucose since bacteria typically utilize glucose. Result (*Blautia obeum*): D‐galactose → Galactose metabolism; arabinose, xylose & glucuronate → Pentose and glucuronate interconversions. Alternatively, check Fructose and mannose metabolism (optional).

8 (Optional) Explore additional carbohydrate‐utilization pathways in *Blautia obeum*. In the summary table, click the Pathway icon to open the *Blautia obeum* pathway collection page. In the Global search box (right side), type “galactose” to locate Galactose metabolism, then click its Pathway icon to open and explore the pathway. Repeat this step for other pathways as needed (e.g., Pentose and glucuronate interconversions and Fructose and mannose metabolism). Result: The galactose pathway indicates that *Blautia obeum* is predicted to convert D‐galactose → galactose‐1‐phosphate → UDP‐galactose + glucose‐1‐phosphate (gal[c] → gal1p[c] → udpgal[c] + g1p[c]), supporting its capacity to utilize galactose as an energy source. Within Pentose and glucuronate interconversions, *Blautia obeum* is predicted to: (i) convert D‐glucuronate to 2‐dehydro‐3‐deoxy‐D‐gluconate 6‐phosphate via D‐mannonate; (ii) convert L‐arabinose to L‐ribulose; and (iii) convert D‐xylose to D‐xylulose. For D‐xylose specifically, *Blautia obeum* is predicted to convert D‐xylose → D‐xylulose → D‐xylulose‐5‐phosphate, which enters the pentose phosphate pathway and is rearranged to fructose‐6‐phosphate and glyceraldehyde‐3‐phosphate, feeding glycolysis and yielding ATP.

## Basic Protocol 4 IDENTIFYING NOVEL DEOXYCHOLIC ACID‐PRODUCING GUT MICROBES

Microbial deoxycholic acid (DCA) is linked to insulin resistance, and *Clostridium scindens* is identified as a key producer via enzymes encoded by the *bai* operon (Wahlström et al., 2024). We hypothesize that other low‐abundance taxa may encode the same 7α‐dehydroxylation capacity but evade detection in metagenomic surveys. This case study shows how to use MicrobiomeNet to explore additional taxa that produce DCA through the 7α‐dehydroxylation pathway. This workflow addresses two questions: (i) which gut microbes (including low‐abundance taxa) are predicted to produce DCA through 7α‐dehydroxylation, and (ii) among those, which exhibit pathway architectures similar to the canonical producer *Clostridium scindens*. The resulting candidate list, taxa‐by‐reaction/pathway matrix, and evidence summary provide mechanistic support for interpreting bile acid–mediated phenotypes and for designing precision interventions (e.g., targeted monitoring, inhibition, or ecological steering of deoxycholic acid producers).

### Necessary Resources

#### Hardware

A computer with internet access

##### Software

An up‐to‐date web browser such as Google Chrome, Mozilla Firefox, or Safari, with JavaScript enabled (see Internet Resources)

##### Files

None

**Figure 12:**
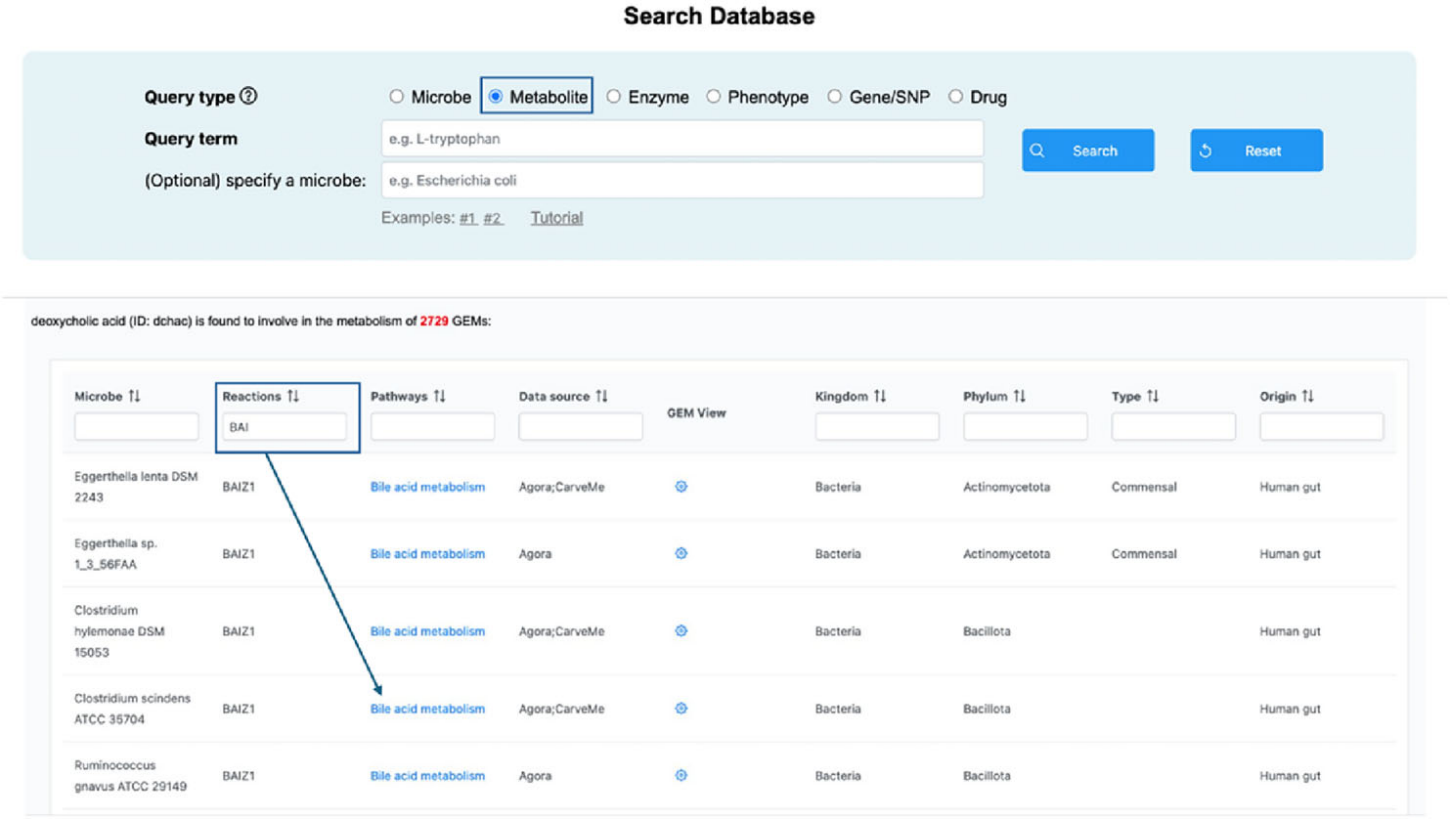
(Top) Searching for deoxycholic acid with a query type “Metabolite”. (Bottom) A summary table of search results for the deoxycholic acid and selection of *bai*‐related reactions.

1 Starting up. Go to the MicrobiomeNet home page (https://microbiomenet.com). In the Search box, set “Query type” to “Metabolite”. Type “deoxycholic acid” as the query term and click the Search button.
2 Exploring the metabolite page and its summary table. In the summary table, locate the Reaction filter box and type “bai” to check for reactions related to the bai operon mentioned in the case study. The table will update to list all bacteria in MicrobiomeNet predicted to produce deoxycholic acid via the BAIZ1 (ID) reaction (Figure 12).

**Figure 13:**
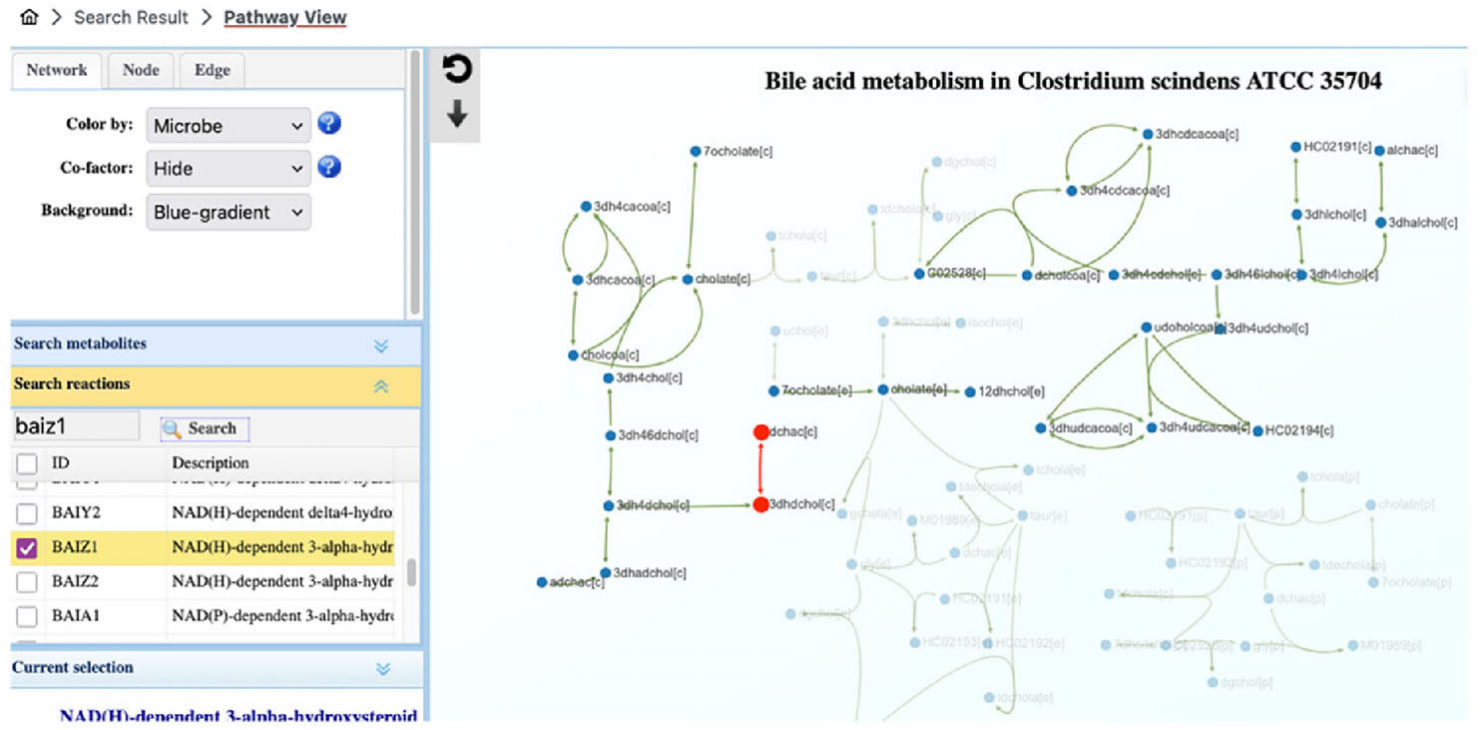
A screenshot of Bile acid metabolism pathway in *Clostridium scindens ATCC 35704*. The highlighted part is the final reaction that produces deoxycholic acid.

3 Understanding the BAIZ1 reaction. At this step, it is important to understand what the BAIZ1 reaction entails and whether it is linked to the *bai* operon of *Clostridium scindens* in the case study. From the summary table, we saw that the BAIZ1 reaction is part of bile acid metabolism; therefore, we should check this pathway. Click on the “Bile acid metabolism” to open the metabolic pathway. On the left panel, select the window “Search reaction” and enter “BAIZ1” and click the Search button to locate the specific reaction in the whole pathway page. The system will highlight the BAIZ1 reaction in red (by default), which converts 3‐dehydrodeoxycholate (3dhdchol[c]) to deoxycholic acid (dchac[c]). 3‐dehydrodeoxycholate (also known as 3‐oxo‐deoxycholic acid) is converted to DCA by enzymes encoded in the *bai* operon (e.g., gene *BaiA2*, the eighth gene in the operon) (Funabashi et al., 2020)
4 Collecting the first list of potential candidates. From the summary page, record the names of all bacteria that produce DCA through the BAIZ1 reaction. *Clostridium scindens* ATCC 35704 should appear in the list.
5 Exploring the DCA‐producing pathway in candidates. From the shortlist, we aim to identify bacteria that produce DCA via the same pathway as *Clostridium scindens* ATCC 35704. From the summary page, click on the “Bile acid metabolism” of *Clostridium scindens* ATCC 35704 to open the Bile acid metabolism view page for this specific bacterium.
6 Locating cholic acid (CA) and deoxycholic acid (DCA) in the pathway. CA is the primary bile acid, which will be converted to DCA by the gut microbiome. To explore this pathway, users first need to locate the CA and DCA in the complex pathway to visualize the reaction series from CA to DCA. Locating the search box (left panel) in the “Search metabolites” window, search for “Cholate” and “deoxycholic”. Note: the system highlights all metabolites matching the text; use the check boxes to select only the exact metabolite. Also, select only the deoxycholic acid with the [c] compartment, as this is where the BAIZ1 reaction takes place (Step 3) (**Figure** **13**).

**Figure 14:**
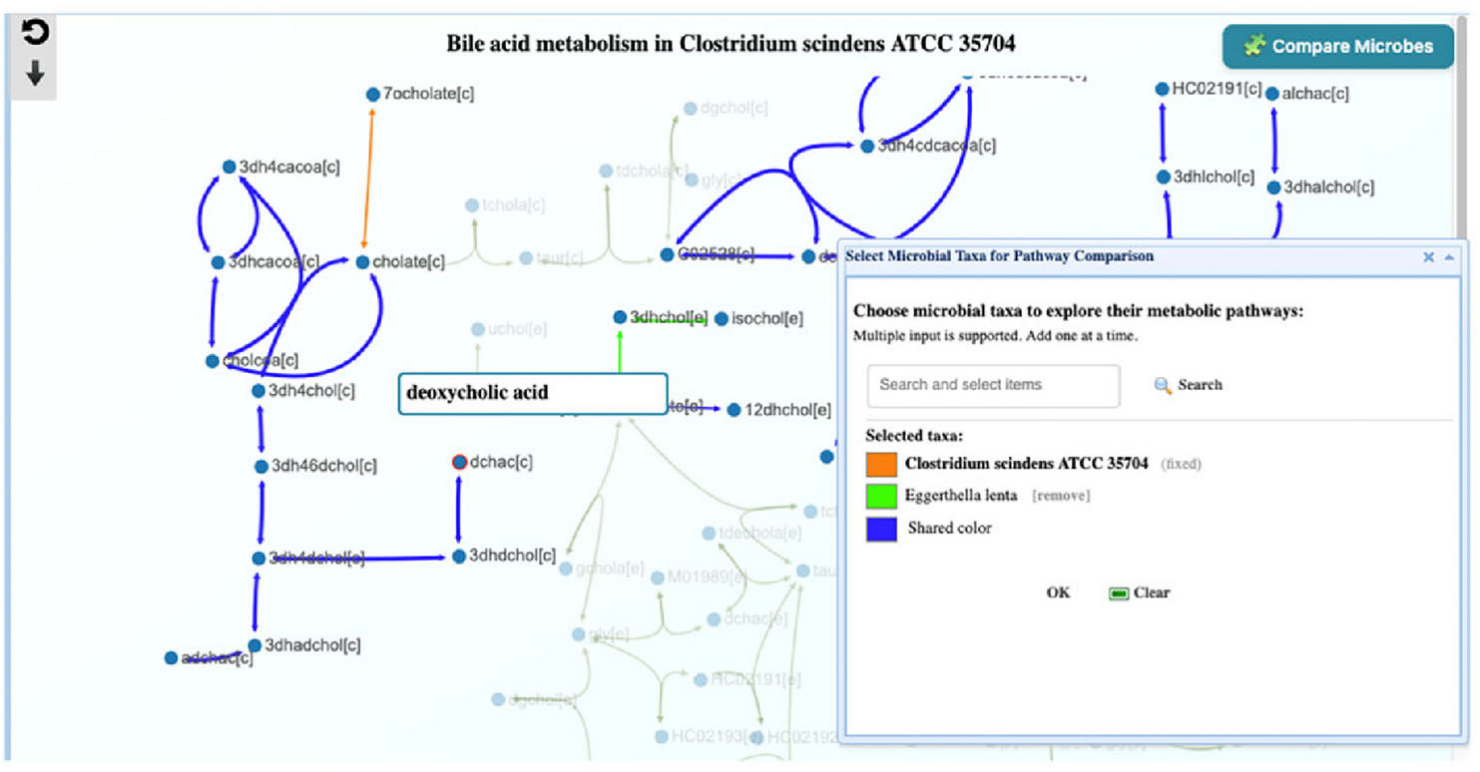
Bile acid metabolism pathway comparison between *Clostridium scindens ATCC 35704* (orange) and *Eggerthella lenta* (Green), where shared pathways are blue. The view focuses on the deoxycholic acid production pathway.

7 Identifying DCA‐producing taxa. To identify DCA‐producing taxa similar to *C. scindens* using the shortlist (Step 4), compare the pathways to see the overlap from CA to DCA between *C. scindens* and other taxa. Using the “Compare microbes” button on the right panel, users can enter another candidate's name (e.g., *Eggerthella lenta*) in the search box of a new floating window (step‐by‐step details can be found in Basic Protocol 1). The results show overlapping CA‐to‐DCA reactions between *C. scindens* and *E. lenta*, suggesting a potential contributor not captured in the case study (**Figure** **14**). Repeat this step for the other taxa in the list.

## Basic Protocol 5 ASSESSING THE FAECALIBACTERIUM PRAUSNITZII–COPROCOCCUS RELATIONSHIP

*F. prausnitzii* and members of the genus *Coprococcus* are prominent butyrate‑producing gut bacteria that have been consistently associated with higher quality of life indicators in human cohorts (Valles‐Colomer et al., 2019). However, their relationship at the mechanistic level remains unclear. It is not clear whether *F. prausnitzii* and *Coprococcus* primarily co‑occur and complement each other's metabolism, or whether they compete for similar substrates and ecological niches. Using MicrobiomeNet, this protocol asks two questions: (1) Is there evidence of association between *F. prausnitzii* and *Coprococcus* across published human cohorts? (2) What is their relationship in the human gut in terms of metabolic capacity and potential ecological overlap, that is, do they appear more compatible (cross‑feeding/co‑existence) or competing for similar resources? This protocol extends that evidence by moving from association to mechanistic interpretation: quantifying inter‐taxon distances, evaluating compatibility versus competition, and inspecting GEM pathways to generate testable, intervention‑oriented hypotheses.

### Necessary Resources

#### Hardware

A computer with internet access

##### Software

An up‐to‐date web browser such as Google Chrome, Mozilla Firefox, or Safari, with JavaScript enabled (see Internet Resources)

##### Files

None

**Figure 15:**
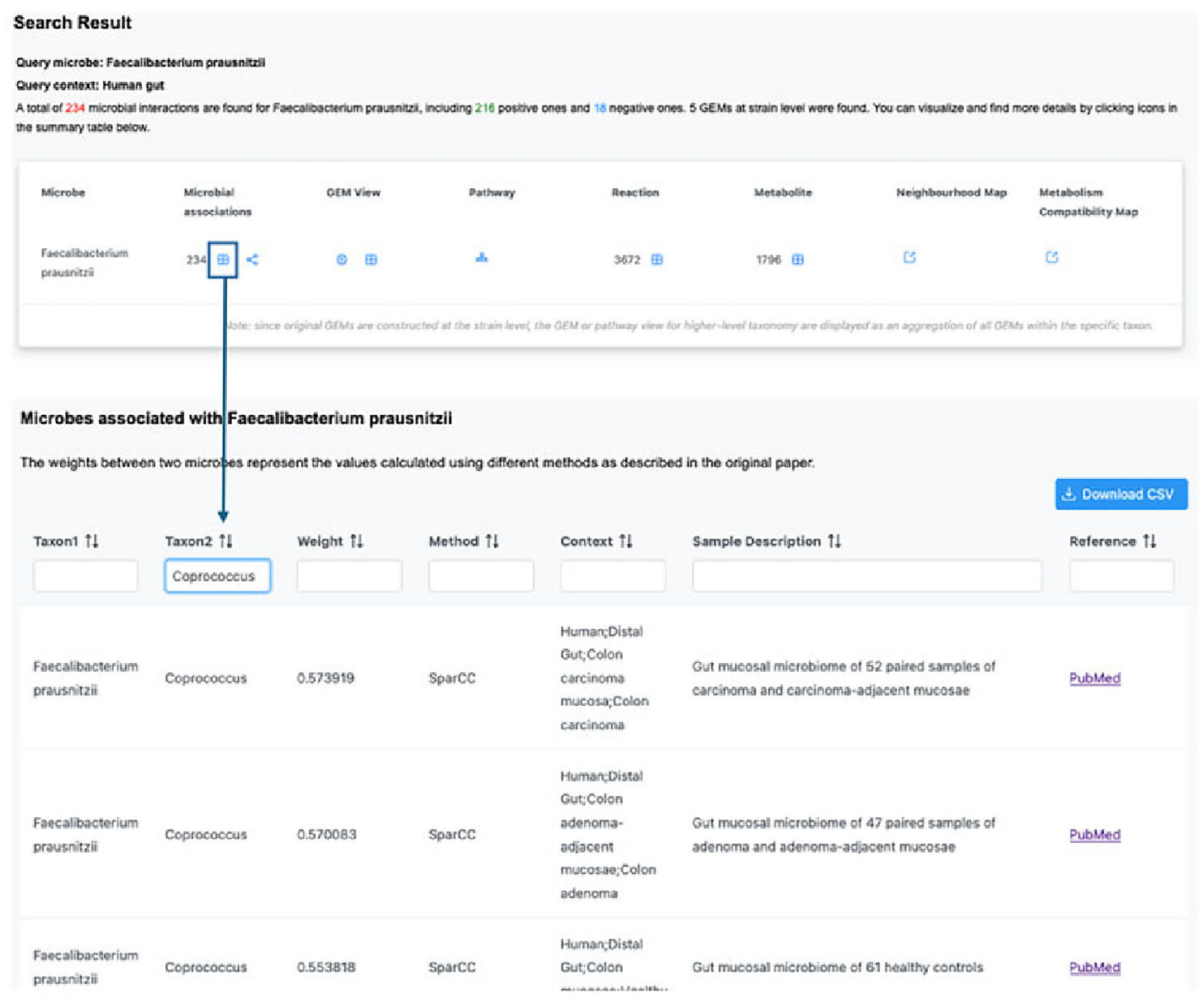
(Top) Summary table of search results for the query taxon *Faecalibacterium prausnitzii* in “human gut” query context. (Bottom) Filtering microbial associations between *Faecalibacterium prausnitzii* and *Coprococcus‐*related taxa.

1 Starting up. Go to the MicrobiomeNet home page (https://microbiomenet.com). In the Search box, set Query type to Microbe (usually selected automatically). Enter *Faecalibacterium prausnitzii* as the Query term, leave “Specify a property” blank, and click the Search button. In the results table, click the microbe name to open the taxon page. From the header, note quick links to Microbial Associations, Neighborhood Map, Metabolism Compatibility Map, Pathways, and Reactions (**Figure** **15**). To address the first question, search MicrobiomeNet's curated association database for direct links between *F. prausnitzii* and *Coprococcus*.

**Figure 16:**
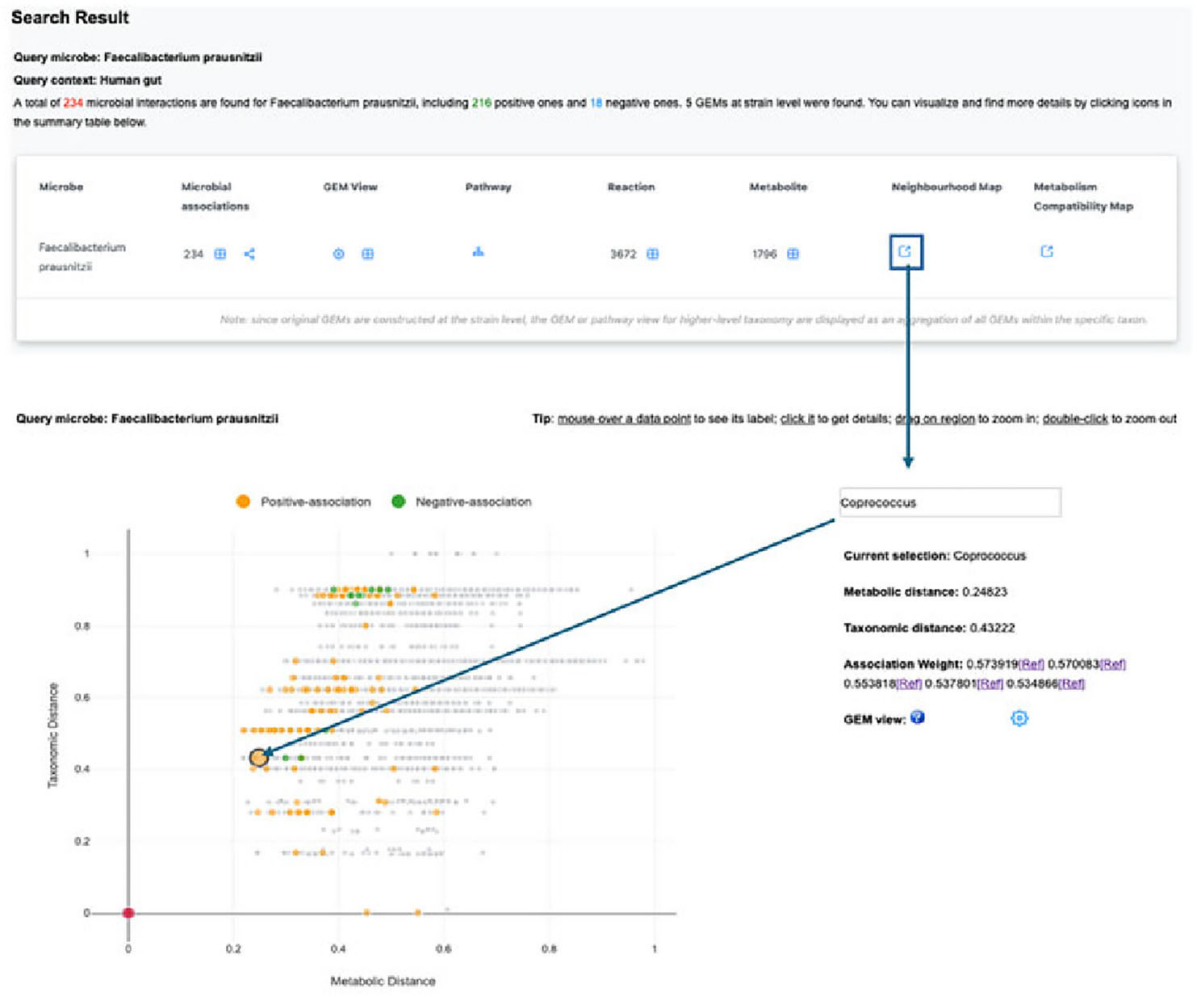
(Top) Selecting neighborhood map from a summary table of *Faecalibacterium prausnitzii* in “human gut” query context. (Bottom) Identification of *the Coprococcus* genus from the *Faecalibacterium prausnitzii* neighborhood map.

2 Identifying Microbial Associations. Locate curated links with *Coprococcus*. On the *F. prausnitzii* page, click the table icon under Microbial Associations. In the Taxon2 filter, type *Coprococcus*. The table shows study‐level correlations between *F. prausnitzii* and *Coprococcus*; record the sign, inference method (e.g., Spearman, SparCC), and study context. Observed in our case study: five entries, all positive correlations, spanning both healthy individuals and colon adenoma cohorts. Note this summary in your results sheet. If species‐level entries exist (e.g., *C. comes*, *C. catus*), record them separately. In the current MicrobiomeNet release, there are five positive associations between *F. prausnitzii* and *Coprococcus* at the genus level and one positive association with *Coprococcus eutactus*, providing direct evidence to help answer the first question (**Figure** **15**).
3 Exploring the Neighborhood Map. To address the second question, first obtain an overview of their metabolic and taxonomic distances using the Neighborhood Map. From the Summary page (Step 1), click Neighborhood Map. In the Search box on the right side of the map, type *Coprococcus*; when the drop‐down menu appears, select *Coprococcus*. In the map, the *Coprococcus* point appears as a larger orange dot (positive association) relative to other points. Click this dot to open the details panel on the right and record: Metabolic distance = 0.248, Taxonomic distance = 0.43, and Association weight = 0.573 (additional weights may be listed if multiple studies contribute). If many points overlap, drag on a region to zoom in; double‐click to zoom out. A metabolic distance of 0.248 suggests that the predicted metabolic functions of *F. prausnitzii* and *Coprococcus* at the genus level are relatively similar rather than highly divergent, but this summary alone cannot tell us whether their interaction is competitive or complementary, so species‐level analysis is needed (Figure 16).

**Figure 17:**
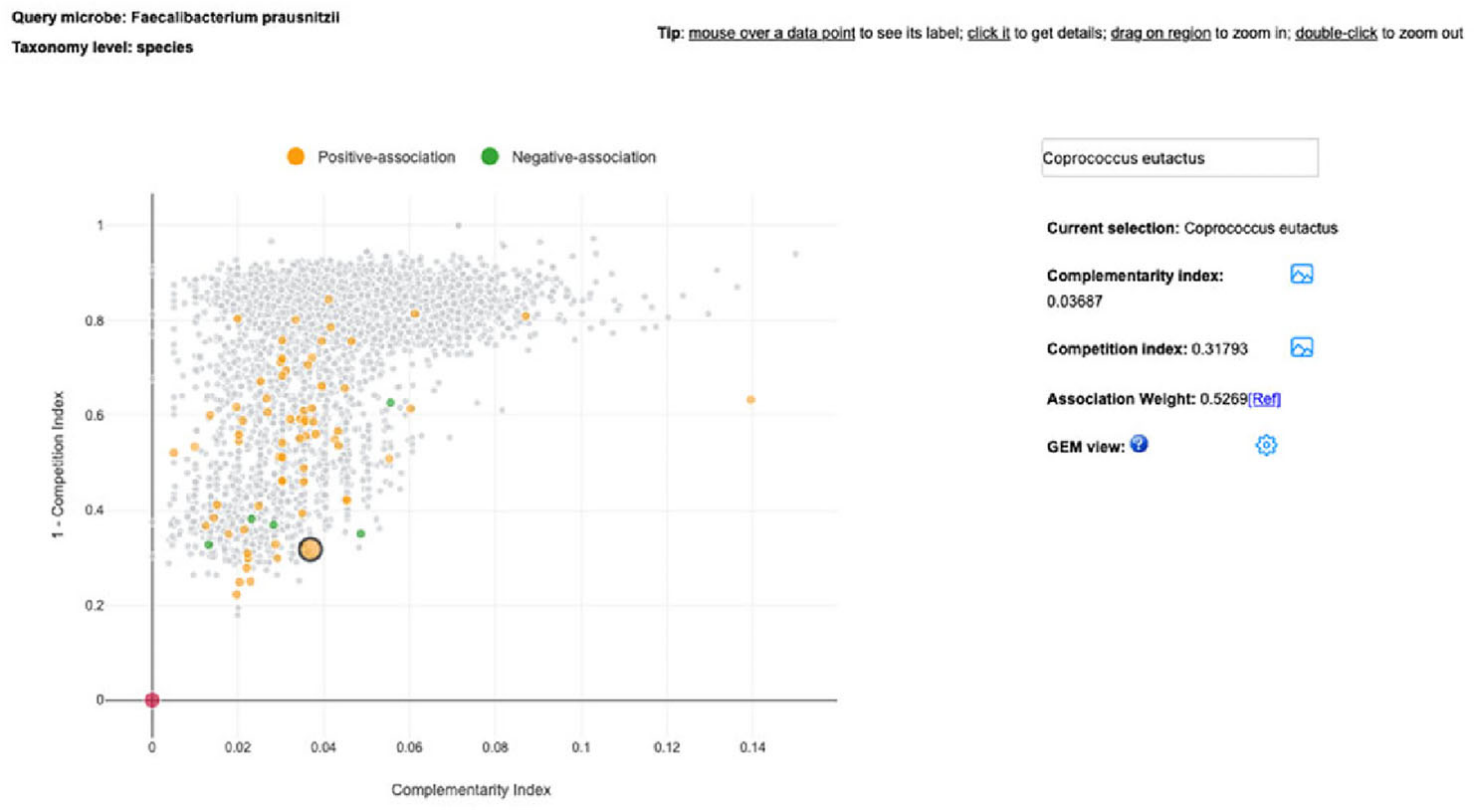
Identification of *Coprococcus eutactus* from *Faecalibacterium prausnitzii* based on the Metabolism Compatibility map.

4 Exploring the Metabolism Compatibility Map. To further explore the second question at the species level, focus on the relationship between *F. prausnitzii* and *C. eutactus*, the only *Coprococcus* species with a curated association identified in Step 2. Return to the Summary page (Step 1) and click the window icon under Metabolism Compatibility Map. In the map, type *Coprococcus* in the search box; the drop‐down will suggest species‐level entries only. Then select *Coprococcus eutactus*. In the map, click the highlighted dot to get the details.
  This map focuses on pairwise species relationships (competition vs. complementarity), so only species are available (**Figure** **17**).

**Figure 18:**
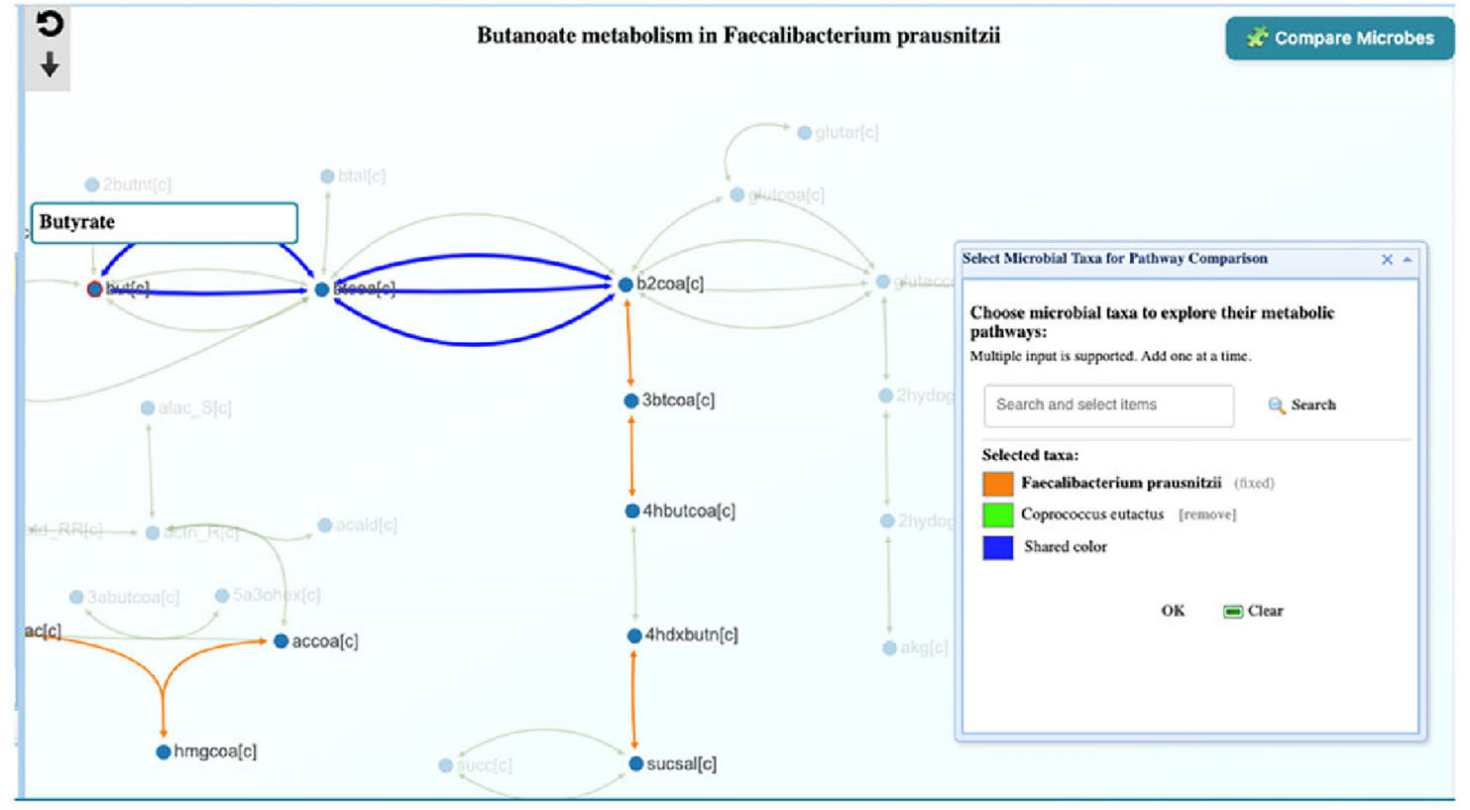
Butanoate metabolism pathway comparison between *Faecalibacterium prausnitzii* (orange) and *Coprococcus eutactus* (Green), where shared pathways are blue. The view focuses on butyrate production.

5 Metabolism compatibility results and interpretation. The result from Step 4 showed that the link between *F. prausnitzii* and *C. eutactus* is positive, with a Complementarity index = 0.036 and a Competition index = 0.68. Taken together, these values indicate low complementarity and substantial niche overlap, meaning that under the current models, *C. eutactus* is more competitive than complementary with *F. prausnitzii*. Positive associations are still plausible if both species respond similarly to the same host or environmental factors. Because both taxa are major butyrate producers, examining their butyrate synthesis pathways helps determine whether they rely on overlapping routes, which can clarify why the compatibility metrics point toward competition.
6 Exploring butyrate (butanoate) metabolism. To support the Step 5 interpretation (more competitive than complementary), examine whether the two taxa use overlapping butyrate‐producing pathways. Return to the Summary page (Step 1) and click the Pathways icon for *F. prausnitzii*. In the Global search box, type Butanoate. The system will surface Butanoate metabolism; click the pathway figure to open the pathway window. Click the “Compare Microbe” button, type *Coprococcus eutactus* in the search box, add in and choose the color, then click “Search” and close the small window. The pathway view now overlays modules present in both *F. prausnitzii* and *C. eutactus*, highlighting shared segments in the butyrate synthesis route(s) (Figure 18). This overlap indicates that the taxa likely exploit similar energy/resource niches, consistent with the low Complementarity index (0.036) and low 1–Competition index (0.31) observed in Step 4 (i.e., relatively high competition).

**Figure 19:**
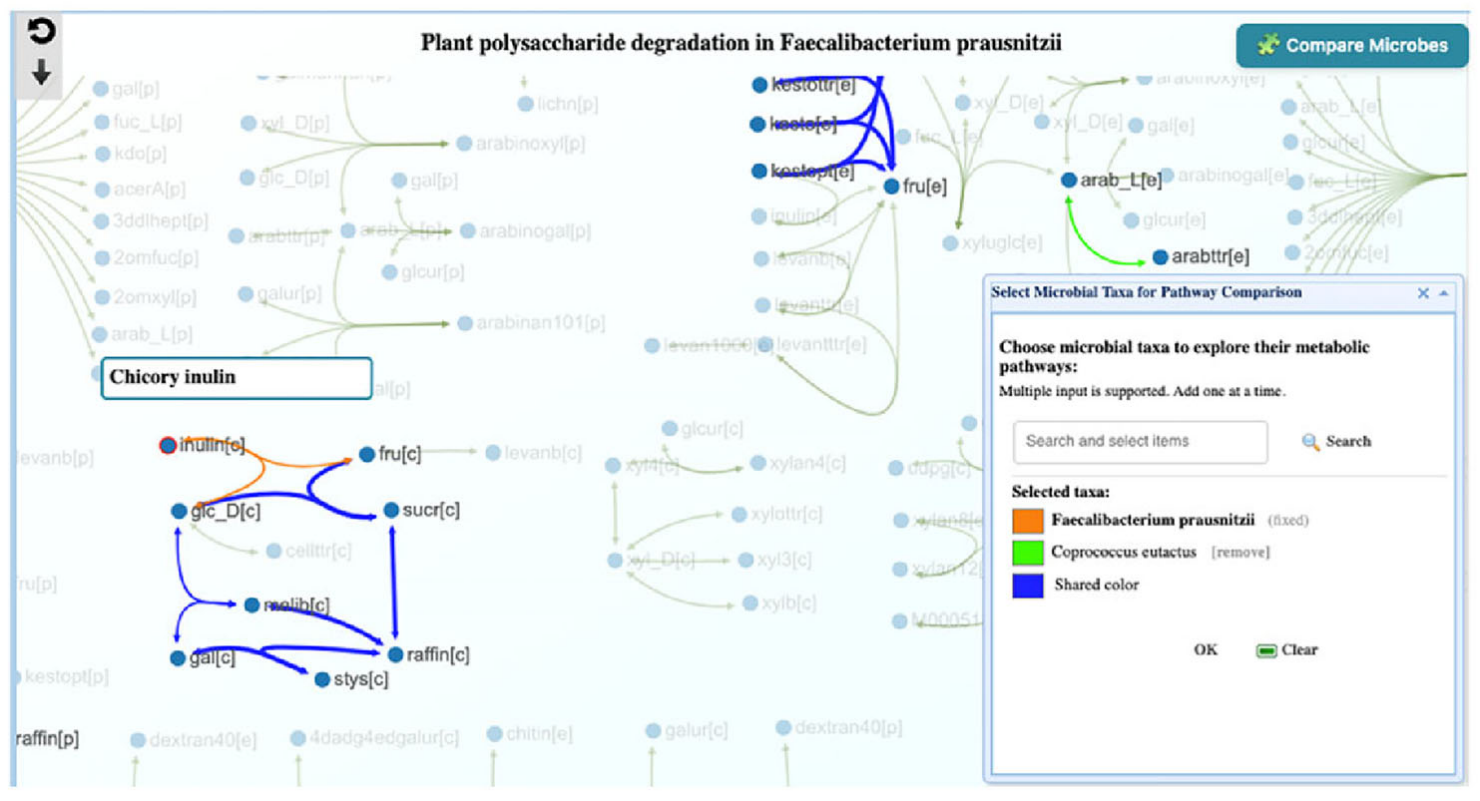
Plant polysaccharide degradation pathway comparison between *Faecalibacterium prausnitzii* (orange) and *Coprococcus eutactus* (Green), where shared pathways are blue. The view focuses on inulin.

7 Exploring plant polysaccharide degradation. Building on this evidence of shared energy niches in butyrate metabolism, we next examine plant polysaccharide degradation to test whether *F. prausnitzii* and *C. eutactus* also compete for the upstream fiber‐derived substrates that fuel butyrate production. From the Summary page (Step 1), click the Pathways icon for *F. prausnitzii*. In the Global search box, type Plant and open Plant polysaccharide degradation. Click the “Compare Microbes” button, type *Coprococcus eutactus*, click search, choose color, and click OK, then close the small window (**Figure** **19**).
  The pathway view shows the unique pathway of F. prausnitzii in orange (as default) and shared pathway in blue. Users can use mouse scroll to zoom into the pathway with Chicory inulin (inulin[c]). It shows that F. prausnitzii can break down inulin into D‐glucose (glc_D[c] and Fructose (fru[c]) while Coprococcus cannot. However, Coprococcus can use these downstream metabolites in other pathways.

## Summary for Protocols 3–5

Addressing the first and second questions posed in the introduction, MicrobiomeNet identifies five positive genus‐level associations and one species‐level link between *F. prausnitzii* and *C. eutactus* across published cohorts. Metabolic distance, compatibility metrics, and pathway overlays reveal that these taxa are metabolically similar and occupy overlapping energy niches. Specifically, the analysis suggests a mechanism of co‐enrichment driven by inulin‐based cross‐feeding: *F. prausnitzii* depolymerizes inulin, releasing fructose and glucose that *C. eutactus* subsequently utilizes. A practical translational step is to evaluate inulin as a candidate fiber to improve mental health outcomes by enriching these butyrate‐producing populations. This approach aligns with emerging microbiota–gut–brain axis research; for instance, inulin‐type fructans and 2’‐fucosyllactose have been shown to alter microbial composition and alleviate stress‐induced mood states (Jackson et al., 2023), while prebiotic intake has been observed to reduce waking cortisol responses and alter emotional bias (Schmidt et al., 2015).

## Commentary

### Background Information

MicrobiomeNet integrates genome‐scale metabolic models with curated microbial associations, enabling users to contextualize observed taxonomic or metabolite changes in mechanistic terms. The resource extends GEM‐based metabolism and links them to study‐level evidence across diverse scenarios.

### Limitations

While microbiome datasets typically operate at the species or genus level, GEMs provide strain‐level resolution. To bridge this gap, MicrobiomeNet aggregates strain‐specific models into composite taxonomic models. This facilitates data integration but may obscure strain‐specific metabolic variations relevant to functional interactions. To reduce false positives, MicrobiomeNet uses a study context based on GEM annotation data and manual curation. Due to metadata limitations, some environmental or host contexts may be incomplete or inaccurate. For practical reasons, GEM visualization excludes very large pathways and disconnected reactions, while retaining complete pathway information. If a specific reaction is not displayed on the central global GEM map, click the pathway link on the left panel to view its detailed visualization. The seed metabolites are estimated based on the curated GEMs, although the true nutrient requirements *in vivo* may not exactly match these predictions. However, our method provides a useful approximation that offers insight into the potential metabolic interactions between species.

### Critical Parameters

Accurate names (e.g., full *Genus or species*) are essential for correct matching to the corresponding GEMs. MicrobiomeNet supports autocomplete to assist user input.

The choice of query type and context determines the analysis perspective and the expected result presentation.

### Troubleshooting

Some common problems and their solutions are summarized in **Table** **3**.

**Table 3:** Troubleshooting

Steps	Potential Issues	Possible Cause	Solution
Step 1 in Protocols 1–5	No results returned	Typographical error or use of abbreviations in the query	Start typing the name and **choose the exact microbe or metabolite from the autocomplete dropdown** that appears; avoid free typing abbreviations
All steps related to pathway/GEM visualization	Pathway/GEM map not shown	Browser cache or outdated browser	Clear cache or refresh browser
All steps related to pathway/GEM visualization	Pathway shows all reactions instead of species‐specific ones	Session expired	Wait a few seconds for the taxon‐specific filtering to finish. Alternatively, click the Refresh icon to reload the view.
All steps related to association network	Association not found	Filters applied or session expired	Double‐check the filter parameters and resubmit. Alternatively, refresh the page.

### Time Considerations

Each basic protocol takes roughly 15–30 min, while a comprehensive exploration of all five protocols may take around 2 hr.

### Understanding Results

Each Basic Protocol produces interactive outputs that serve as benchmarks for correct execution and as examples for biological interpretation. Figures/tables cited in the protocol steps should reproduce the features described below if the workflow has been followed correctly.

#### Basic Protocol 1

This protocol guides users in characterizing the detailed functional profile of a specific species, using *F. prausnitzii* as a case study. Users will explore metabolic capabilities across metabolite, reaction, and pathway levels, specifically visualizing the mechanism of butyrate production via Butanoate metabolism. Furthermore, the protocol demonstrates how to apply standard and weighted Jaccard indices to quantitatively assess metabolic similarities between a specific taxon and the community, or between microbial pairs.

#### Basic Protocol 2

This protocol guides users in interpreting known associations among microbes or in assessing whether potential metabolic interactions exist. Using *F. prausnitzii* as an example, users should retrieve its association network, including the reported relationship with *B. fragilis*, in the context of a healthy gut. The information collected from the literature, as shown in the table or network, indicates a moderate statistical correlation between these two species, while the Metabolism Compatibility Map shows that their complementarity index is among the highest among all species paired with *F. prausnitzii*. Users can further visualize their potential interactions at both the level of the entire metabolic network and within specific metabolic pathways, such as Butanoate metabolism. This observation is consistent with previous studies showing that *B. fragilis* can produce substrates used by *F. prausnitzii*, supporting a biologically plausible cross‐feeding relationship.

#### Basic Protocol 3

Users will obtain a pathway‐level map that summarizes plant polysaccharide‐degrading modules across the selected taxa. In the arabinoxylan example, *Bifidobacterium longum* contains arabinoxylan degradation steps, whereas *Blautia obeum* lacks those steps but encodes routes to utilize the released monosaccharides. This complementary layout explains their co‐response in the fiber arm. Minor differences in reaction coverage across GEM versions may occur, but should not alter the overall complementary pattern. Practically, this means *B. longum* acts as the primary depolymerizer of arabinoxylan, while *B. obeum* consumes the resulting sugars—consistent with cross‐feeding rather than competition. MicrobiomeNet links these pathway features to observed co‐increases and guides choices, such as fiber type/dose or fiber pairing, to enrich a desired consortium.

#### Basic Protocol 4

A correctly executed workflow yields a prioritized list of candidate DCA producers via the BAIZ1 7α‐dehydroxylation step. Pathway overlays anchored on *Clostridium scindens* then identify taxa that share the CA→DCA sub‐pathway; some candidates fully overlap with *C. scindens*, while others show partial overlap. In this case study, the final list includes *Eggerthella lenta*, *Clostridium hylemonae*, [*Clostridium*] *hiranonis*, and *Lachnospiraceae bacterium 5_1_57FAA*—candidates likely to contribute materially to DCA pools under the relevant conditions. By combining a metabolite→reaction (deoxycholic acid→BAIZ1) query with pathway overlays, MicrobiomeNet systematically nominates additional low‐abundance 7α‐dehydroxylators and prioritizes those with *Clostridium scindens*–like pathway architecture, addressing the limitation noted by (Wahlström et al., 2024) and converting suspected but unidentifiable contributors into a concrete, testable candidate set for targeted monitoring or ecological steering of DCA producers.

#### Basic Protocol 5

This case study returns two main outputs—curated association summaries across published cohorts and pathway overlays for butyrate production and plant polysaccharide degradation—that together show that *F. prausnitzii* and *Coprococcus* are consistently positively associated, share key butyrate‐linked functions, and differ in how they access fiber‐derived substrates. In the plant polysaccharide degradation pathway, *F. prausnitzii* carries modules to depolymerize inulin, whereas *C. eutactus* lacks upstream inulin depolymerization but encodes uptake and catabolism of the breakdown products (e.g., fructose and glucose). This pattern supports an inulin‐based cross‐feeding relationship in which *F. prausnitzii* acts as a primary degrader, and *C. eutactus* consumes the released simple sugars and, together with the low complementarity and substantial niche overlap suggested by the compatibility indices, points to partial division of labor on inulin superimposed on competition for some downstream energy sources.

### Author Contributions

J.X. conceptualized the project and supervised the study. Y.L. and J.X. developed the MicrobiomeNet platform. Y.L. drafted Protocols 1 and 2, while K.N.N. drafted Protocols 3 through 5. All authors read and approved the final manuscript.

### Conflict of Interest

None declared.

## Data Availability

Data sharing not applicable to this article as no datasets were generated or analyzed during the current study
